# Enterovirus infections in pediatric patients hospitalized with acute gastroenteritis in Chiang Mai, Thailand, 2015–2018

**DOI:** 10.7717/peerj.9645

**Published:** 2020-08-17

**Authors:** Kitsakorn Rojjanadumrongkul, Kattareeya Kumthip, Pattara Khamrin, Nuthapong Ukarapol, Hiroshi Ushijima, Niwat Maneekarn

**Affiliations:** 1 Department of Microbiology, Chiang Mai University, Faculty of Medicine, Chiang Mai, Thailand; 2 Emerging and Re-emerging Diarrheal Viruses Cluster, Chiang Mai University, Chiang Mai, Thailand; 3 Department of Pediatrics, Chiang Mai University, Faculty of Medicine, Chiang Mai, Thailand; 4 Department of Developmental Medical Sciences, The University of Tokyo, School of International Health, Graduate School of Medicine, Tokyo, Japan; 5 Department of Pathology and Microbiology, Nihon University School of Medicine, Tokyo, Japan

**Keywords:** Gastroenteritis, Diarrhea, Enterovirus, Genotyping, Children, Thailand

## Abstract

**Background:**

Infection with viruses especially rotavirus, norovirus, astrovirus, and adenovirus has been known to be a major cause of acute gastroenteritis in children under 5 years of age globally, particularly in developing countries. Also, some genotypes of enteroviruses (EVs) have been reported to be associated with gastroenteritis. This study is aimed to investigate the prevalence and genotype diversity of EV in children admitted to hospitals with acute gastroenteritis.

**Methods:**

A total of 1,736 fecal specimens were collected from children hospitalized with diarrhea in Chiang Mai, Thailand from 2015 to 2018. All specimens were tested for the presence of EV by RT-PCR of the 5′ untranslated region. The genotypes of EV were further identified by nucleotide sequencing and phylogenetic analysis of the viral protein 1 (VP1) gene.

**Results:**

EV was detected in 154 out of 1,736 specimens (8.9%) throughout the study period. The prevalence of EV detected in 2015, 2016, 2017, and 2018 was 7.2%, 9.0%, 11.2%, and 8.6%, respectively. EV was detected all year round with a high prevalence during rainy season in Thailand. Overall, 37 genotypes of EV were identified in this study. Among these, coxsackievirus (CV)-A24 and CV-B5 (7.5% each), and EV-C96 (6.8%) were the common genotypes detected.

**Conclusion:**

This study demonstrates the prevalence, seasonal distribution, and genotype diversity of EV circulating in children hospitalized with acute gastroenteritis in Chiang Mai, Thailand during the period 2015 to 2018.

## Introduction

Acute gastroenteritis is one of the most typical causes of morbidity and mortality in humans of all age groups with the highest incidence in children in the age range of 6–24 months (Dennehy, 2011), particularly, in low-income and middle-income countries. Almost every child around the world experiences diarrhea during the first 5 years of life. Diarrhea accounts for 25–30% of all deaths in children in this age group worldwide. Each year, acute gastroenteritis accounts for 3–5 billion cases and nearly 2 million deaths of children globally (King et al., 2003). In developed countries, it is also a common reason for admission to hospital. The cost of gastroenteritis is high and the disease remains an important health issue (Elliott, 2007). Microbial infection is the common cause of acute gastroenteritis, especially the virus is responsible for more than 70% of diarrhea cases, followed by bacteria (10–20%) and protozoa (less than 10%) (Elliott, 2007). Viruses that are the most common cause of acute gastroenteritis include rotavirus, calicivirus, adenovirus, and astrovirus (Desselberger, 2017). However, enteroviruses have also been recently associated with acute gastroenteritis (Rao et al., 2013; Patil, Chitambar & Gopalkrishna, 2015).

Human enteroviruses (EVs) are a member of the *Enterovirus* genus in the *Picornaviridae* family. The Enterovirus genus comprises 15 species, including 12 EV species (EV-A to EV-L) and 3 rhinovirus species (RV-A to RV-C). EVs which can infect human are currently classified into 4 species, including EV-A, EV-B, EV-C, and EV-D. The particle of EV is a spheroidal, non-enveloped virion with 22–30 nm in diameter. The viral genome is a single-stranded RNA molecule of 7,389 to 7,441 nucleotides long that is packed within an icosahedral capsid (Van der Linden, Wolthers & Van Kuppeveld, 2015).

Although most EV infections are commonly asymptomatic, they have been reportedly associated with several diseases, for example, acute flaccid paralysis, aseptic meningitis, encephalitis, acute hemorrhagic conjunctivitis, hand-foot-and-mouth disease, and diarrhea (Pons-Salort, Parker & Grassly, 2015; Tapparel et al., 2013).

An association between EV infection and diarrheal disease has been documented in several reports conducted in several countries, including Thailand, Italy, Japan, Vietnam, Djibouti, and India (Chaimongkol et al., 2012; Maslin et al., 2007; Patil, Chitambar & Gopalkrishna, 2015; Pham et al., 2010; Phan et al., 2005; Rao et al., 2013; Rovida et al., 2013; Shen et al., 2019; Silva et al., 2008). These studies reported the detection of EV in fecal samples from patients with acute gastroenteritis. The prevalence of EV infection reported from these studies was different depending on geographical study regions with a range of 1.2–42%. In Thailand, the epidemiological data of EV infection in association with diarrhea and information about the genotypic diversity of EV are still limited and not conducted continuously. Therefore, this study aimed to investigate the prevalence and to perform molecular characterization of EVs circulating in children with diarrhea in Thailand from 2015 to 2018.

## Material and Methods

### Specimen collection

A total of 1,736 fecal specimens were collected from children hospitalized with acute gastroenteritis at four major hospitals in Chiang Mai Province, Thailand, including Maharaj Nakorn Chiang Mai hospital, Nakorn Ping Hospital, Sanpatong Hospital, and Rajavej Chiang Mai Hospital. The study period started from January 2015 to December 2018. The ages of these patients ranged from neonate up to 5 years old. The criteria of acute gastroenteritis are defined as the sudden passage of watery stools (at least three times per day) for a duration of fewer than 2 weeks (Bassetto et al., 2019). All fecal specimens were suspended in phosphate-buffered saline, pH 7.4 for viral genome extraction. This study was approved by the Research Ethics Committee of the Faculty of Medicine, Chiang Mai University (MIC-2561-05659). The written informed consent form was obtained from parents before collecting samples from their children.

### Detection of EV in fecal specimens

Viral genomic RNA was extracted from 200 µl of the supernatant of 10% fecal suspension using the Geneaid Viral Genome Extraction Kit (Geneaid, Taipei, Taiwan) according to the manufacturer’s instruction. The viral RNA genome was then subsequently reverse transcribed by reverse transcriptase enzyme according to the manufacturer’s protocol (Invitrogen, Carlsbad, USA). In some cases, the viral genome was kept at −80 °C until tested. Polymerase chain reaction (PCR) amplification of 5′ untranslated region (5′ UTR) of EV genome was performed using EV-specific primers, sense primer F1 (5′-CAAGCACTTCTGTTTCCCCGG-3′) and antisense primer R1 (5′-ATTGTCACCATAAGCAGCCA-3′). To generate the PCR product size of 440 base pairs (Zoll et al., 1992), the amplification was performed under the following thermal cycling conditions. First, 95 °C for 3 min, followed by 35 cycles of 94 °C for 1 min, 50 °C for 1 min, 72 °C for 1 min and the final extension at 72 °C for 10 min. To identify the enterovirus genotype, the EV genome was further amplified for partial viral protein 1 (VP1) region as described previously (Nix, Oberste & Pallansch, 2006; Oberste et al., 2003). The full length of enteroviruses VP1 region ranged from 834 to 951 nucleotides long. Based on Nix and Oberste’s protocol, nested-PCR amplification was performed with outer primers 224 (5′-GCIATGYTIGGIACICAYRT-3′) and 222 (5′-CICCIG GIGGIAYRWACAT-3′), and inner primers AN89 (5′-CCAGCACTGACAGCAGYNGARAYNGG-3′) and AN88 (5′-TACTGGACCACCTGGNGGNAYRWACAT-3′). Thermal cycling condition of the first-round PCR was as followed, 95 °C for 3 min, followed by 35 cycles of 95 °C for 1 min, 42 °C for 45 s, 72 °C for 1 min and the final extension at 72 °C for 10 min. The second-round PCR amplification was then performed using thermal cycling condition as the following, 95 °C for 3 min followed by 40 cycles of 95 °C for 1 min, 55 °C for 45 s, 72 °C for 40 s and the final extension at 72 °C for 10 min. The amplicon sizes of the first and the second-round PCR products were 993 and 350 base pairs, respectively.

### Nucleotide sequencing and phylogenetic analysis

The PCR products of the VP1 gene of human EV were purified by Gel/PCR DNA Fragments Extraction Kit (Geneaid, Taipei, Taiwan) according to the manufacturer’s protocol. Nucleotide sequencing of the purified PCR products was performed by a fluorescence-based cycle sequencing method using the BigDye® Terminator Cycle Sequencing Kit (Applied Biosystem, Carlsbad, USA). The obtained nucleotide sequences of the partial VP1 gene were assembled and analyzed manually by MEGA (Version X) software (https://www.megasoftware.net). Multiple sequence alignment was performed using MEGA (Version X). Because of different enterovirus genotypes possess different lengths of VP1 region, therefore, the length of VP1 nucleotide sequences used for phylogenetic analysis varied depending on enterovirus species. The VP1 nucleotide sequences of enterovirus species A varied from 219 to 225 nucleotides. Enterovirus species B, C, D and RV-A varied from 237–255 nucleotides, 258–267 nucleotides, and 264–312 nucleotides, respectively. The reference sequences were obtained from the NCBI GenBank database by using the BLAST server (https://blast.ncbi.nlm.nih.gov/Blast.cgi). Phylogenetic trees of the partial VP1 gene of EV were conducted by MEGA (version X) software using the neighbor-joining method (Kumar et al., 2018). A bootstrap analysis (*n* = 1,000) was performed.

### Nucleotide accession number

Nucleotide sequences of the partial VP1 gene of EV strains reported in this study have been deposited in the GenBank database under the accession numbers MN699137 –MN699283 (Files S2 and S3).

## Results

### Prevalence and seasonal distribution of EV

**Figure 1:**
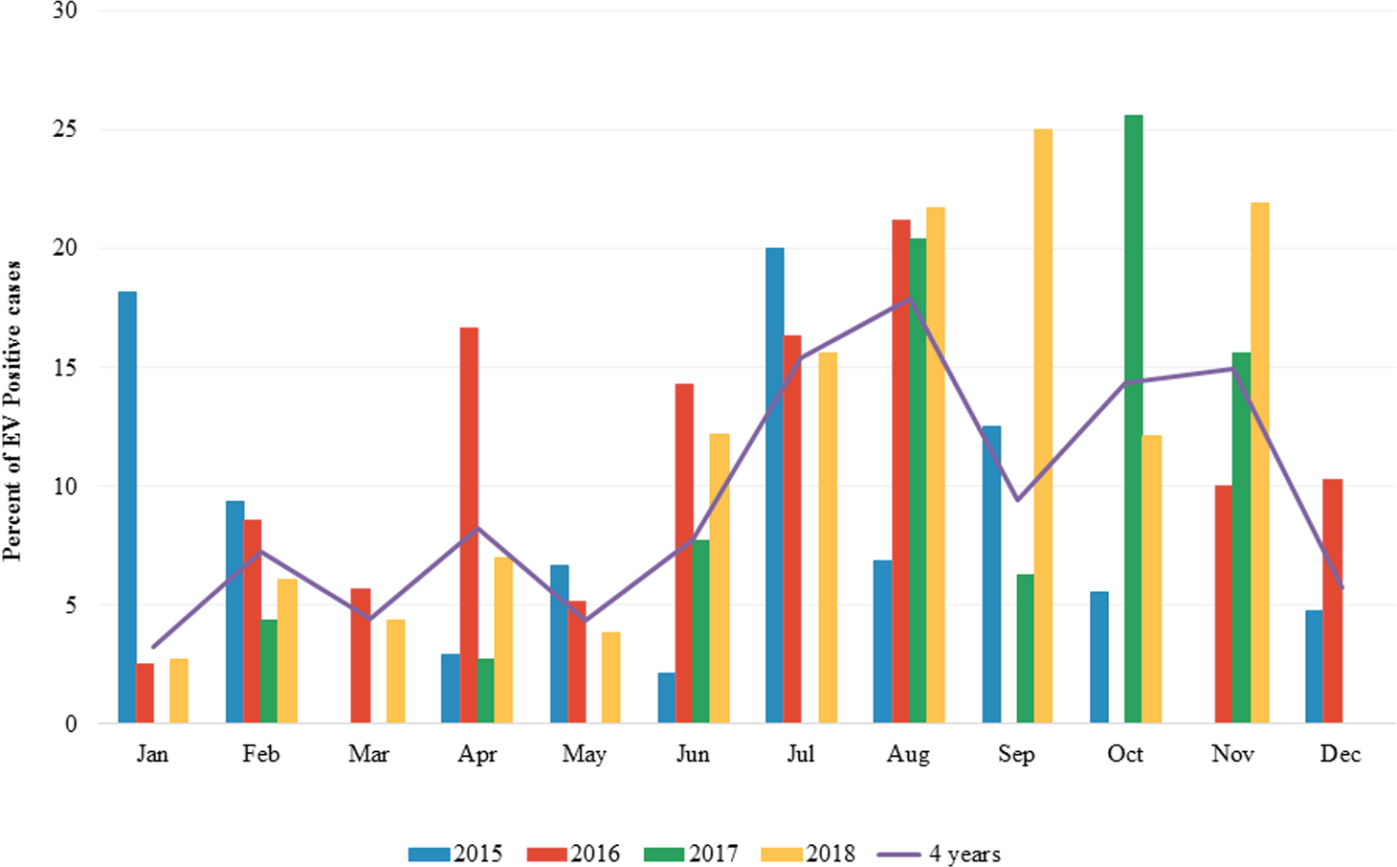
Monthly distribution of enterovirus infection 2015 to 2018.

The overall prevalence of EV infection in children with diarrhea during the 4 years study period was 8.9% (154 of 1,736) ranging from 7.2–11.2%. In 2015, 24 out of 335 samples were positive for EV (7.2%). In 2016, 46 out of 509 samples were positive for EV (9.0%). In 2017, 31 out of 278 specimens were positive for EV (11.2%) while in 2018, 53 out of 614 samples were positive for EV (8.6%) (Table 1). The seasonal distribution of EV infection was investigated from January 2015 to December 2018 (Fig. 1 and File S1). During the 4-year study period, EV was detected throughout the year with a high prevalence (9.43–17.91%) in rainy season (Jul-Aug-Sep) and beginning of winter (Nov) in Thailand. The highest detection rate was in August (17.91%).

**Table 1:** Prevalence of enterovirus infection in children with acute gastroenteritis in Chiang Mai, Thailand 2015–2018.

Year	Number of specimens tested	Number of EV positive specimens (%)
2015	335	24 (7.2%)
2016	509	46 (9.0%)
2017	278	31 (11.2%)
2018	614	53 (8.6%)
**Total**	**1,736**	**154 (8.9%)**

### Genotype distribution of EV

The EV-positive samples were identified for the virus genotypes by nucleotide sequence analysis of the partial VP1 region. From 154 EV positive cases, 147 EV strains could be successfully amplified for the VP1 gene. Human EV species A, B, C, D and rhinovirus (RV) species A were detected in this study. Of 147 EV isolates, 32 out of 147 samples (21.7%) were EV-A, 65 (44.2%) were EV-B, 47 (32%) were EV-C, 1 (0.7%) was EV-D, and 2 (1.4%) were RV-A (Table 2).

The genotypic distribution of EV detected in this study is shown in Table 2. Overall, 37 EV genotypes were detected in this study. The most common genotype detected was coxsackievirus (CV)-A24 and CV-B5 (7.5% each), followed by EV-C96 (6.8%), poliovirus (PV)-3 (6.1%), CV-A4, and CV-A13 (each 5.4%). The EV species A detected in this study included 8 different genotypes of CV-A2, CV-A4, CV-A5, CV-A6, CV-A8, CV-A10, CV-A16, and EV-A71. The species B was comprised of 19 different genotypes, including echovirus (E) 1, E3, E5, E6, E9, E13, E14, E15, E16, E18, E19, E21, E25, E30, CV-A9, CV-B1, CV-B3, CV-B4, and CV-B5. The species C included 7 genotypes of CV-A13, CV-A19, CV-A21, CV-A24, EV-C96, PV-2, and PV-3. Species D, only one genotype of EV-D68 was detected. The RV detected in this study was species A which included RV-A81 and RV-A82 genotypes.

### Phylogenetic analysis of the EV-VP1 gene

**Figure 2:**
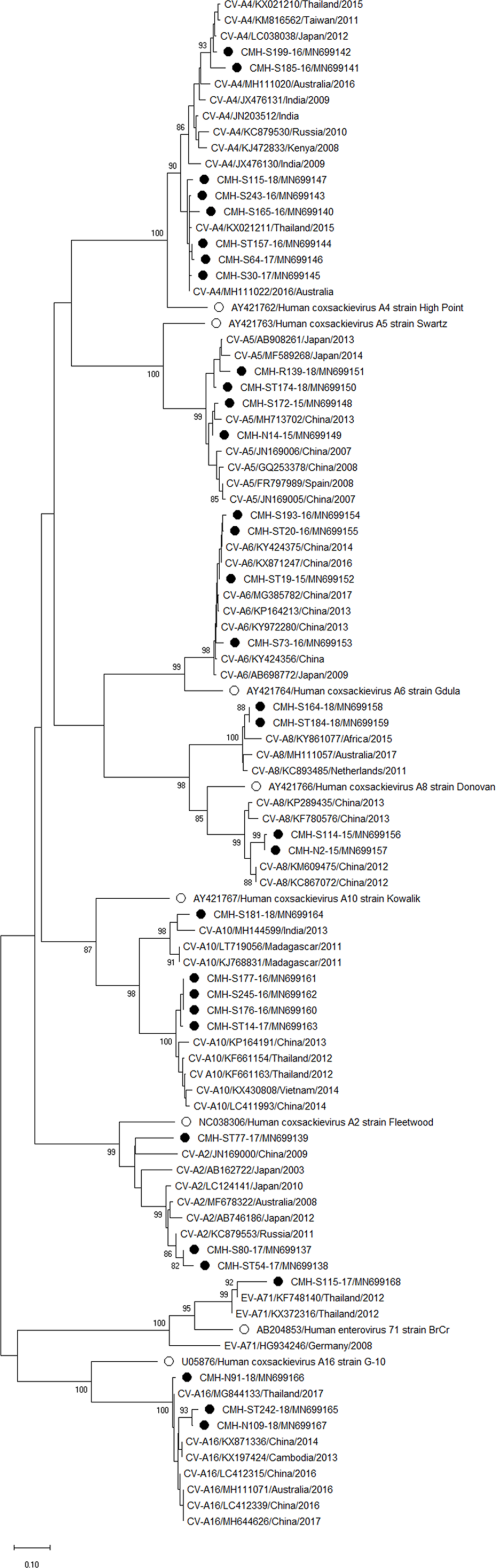
Phylogenetic analysis of the partial nucleotide sequence of VP1 region (219–225 nucleotides) of enterovirus A in this study (•), prototype strains (∘), and GenBank database (reference strains).

**Figure 3:**
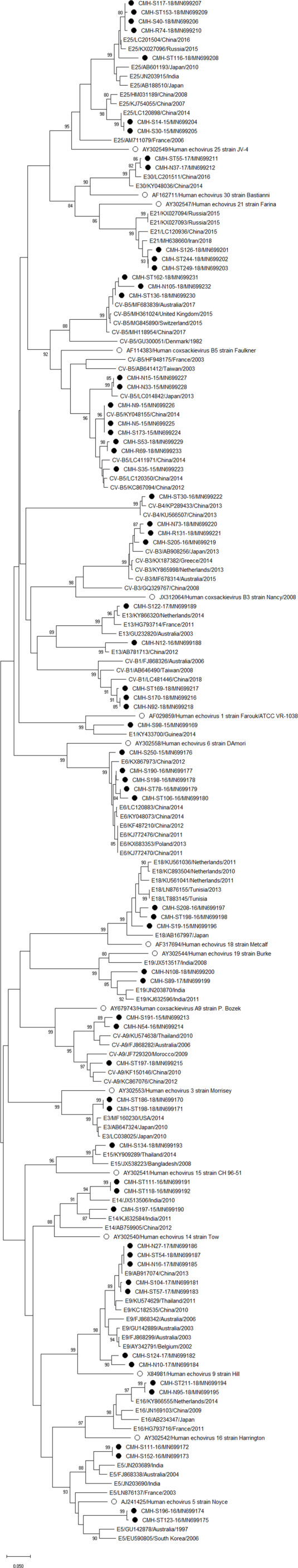
Phylogenetic analysis of the partial nucleotide sequence of VP1 region (237–255 nucleotides) of enterovirus B in this study (•), prototype strains (∘), and GenBank database (reference strains).

**Figure 4:**
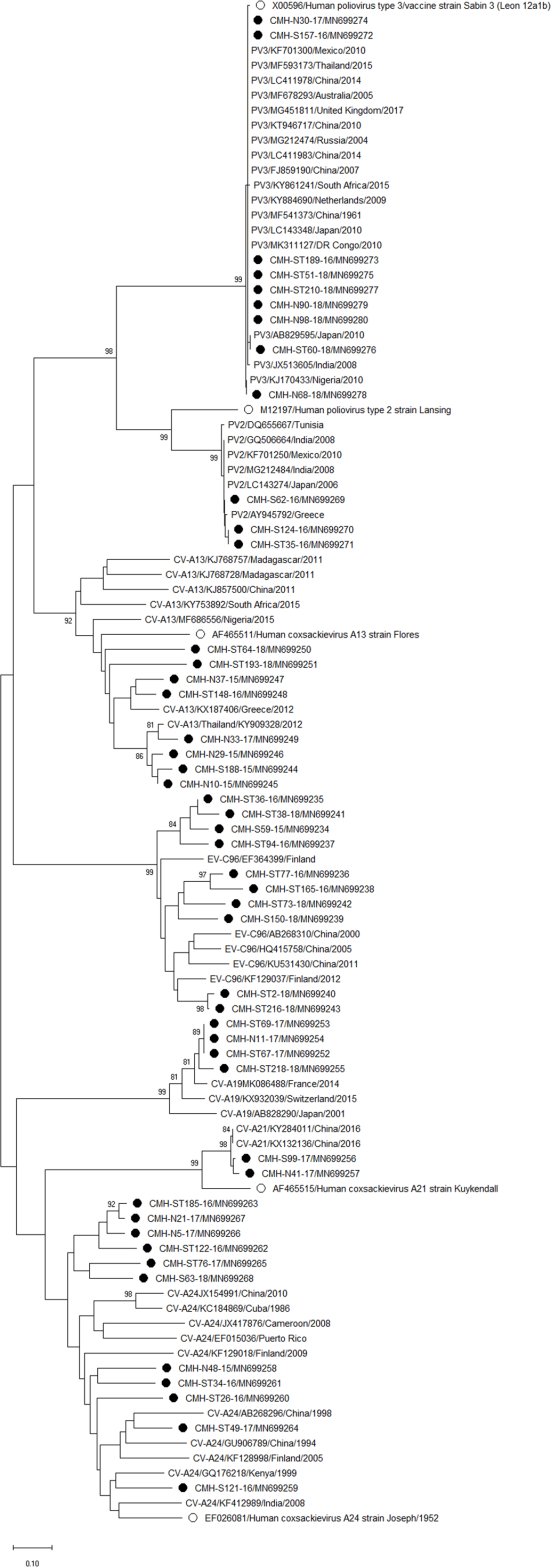
Phylogenetic analysis of the partial nucleotide sequence of VP1 region (258–267 nucleotides) of enterovirus species C in this study (•), prototype strains (∘), and GenBank database (reference strains).

**Figure 5:**
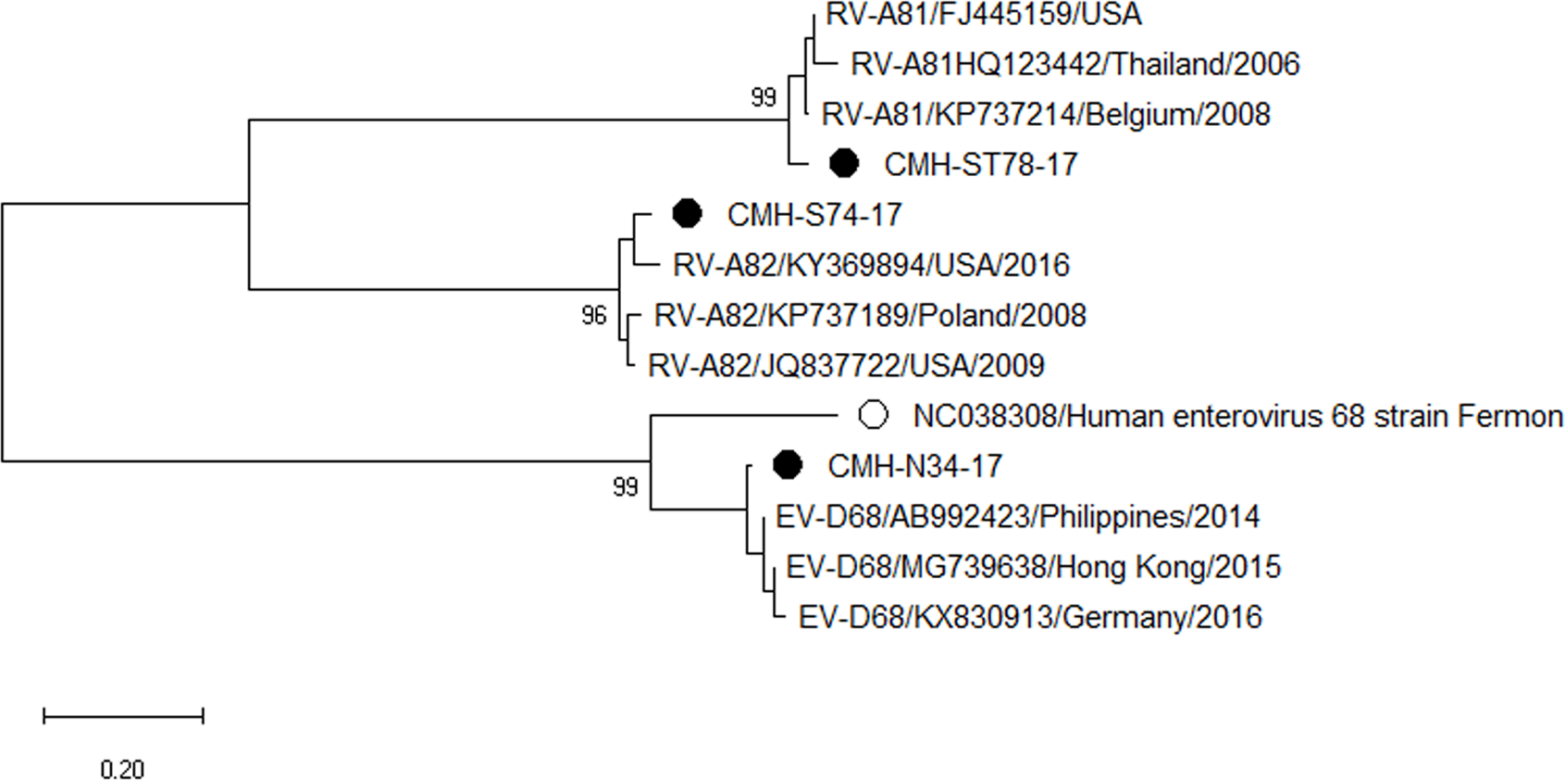
Phylogenetic analysis of the partial nucleotide sequence of VP1 region (264–312 nucleotides) of enterovirus D and rhinovirus A in this study (•), enterovirus D prototype strain (∘), and GenBank database (reference strains).

Phylogenetic trees of the VP1 sequences of EV-A, EV-B, EV-C, and EV-D together with RV-A detected in this study are shown in Figs. 2, 3, 4 and 5, respectively. The EV species A , B, and C detected in this study were closely related to entetovirus strains reported previously worldwide, including several countries in Asia, Europe, Africa, America, and Australia. The same EV genotypes were grouped together separated from other genotypes. One strain of EV-D68 detected in this study was closely related to the EV-D68 reference strains reported previously from the Philippines, Hong Kong, and Germany, sharing 96.5–99.3% nucleotide sequence identities.The RV-A species included RV-A81 and RV-A82 genotypes which were closely related to the reference strains reported from Thailand, USA, Belgium, and Poland, with 93.7–98.1% nucleotide sequence identities.

**Table 2:** Distribution of enterovirus genotypes detected in children hospitalized with acute gastroenteritis 2015–2018.

**Year**	**EV-A (*n* = 32)**								**EV-B (*n* = 65)**																			**EV-C (*n* = 47)**							**EV-D**	**RV**	
	CV A2	CV A4	CV A5	CV A6	CV A8	CV A10	CV A16	EV A71	E1	E3	E5	E6	E9	E13	E14	E15	E16	E18	E19	E21	E25	E30	CV A9	CV B1	CV B3	CV B4	CV B5	EV C96	CV A13	CV A19	CV A21	CV A24	PV2	PV3	EV D68	RV A81	RV A82
**2015**	–	–	2	1	2	–	–	–	1	–	–	1	–	–	1	–	–	1	–	–	2	–	1	–	–	–	6	1	4	–	–	1	–	–	–	–	–
**2016**	–	5	–	3	–	3	–	–	–	–	4	4	–	1	2	–	–	2	–	–	–	–	1	–	1	1	–	4	1	–	–	5	3	2	–	–	–
**2017**	3	2	–	–	–	1	–	1	–	–	–	–	6	1	–	–	–	–	1	–	–	2	–	–	–	–	–	–	1	3	2	4	–	1	1	1	1
**2018**	–	1	2	–	2	1	3	–	–	2	–	–	1	–	–	1	2	–	1	3	5	–	1	3	2	–	5	5	2	1	–	1	–	6	–	–	–
**4 years**	3	8	4	4	4	5	3	1	1	2	4	5	7	2	3	1	2	3	2	3	7	2	3	3	3	1	11	10	8	4	2	11	3	9	1	1	1

## Discussion

The prevalence of EV infection in fecal specimens from patients with acute gastroenteritis have been reported worldwide with a wide range of infection rate from 1.2 to 42% (Chaimongkol et al., 2012; Maslin et al., 2007; Patil, Chitambar & Gopalkrishna, 2015; Pham et al., 2010; Phan et al., 2005; Rovida et al., 2013; Shen et al., 2019; Silva et al., 2008). In Thailand, only 3 epidemiological studies of EV infection in children with acute gastroenteritis have been reported so far. The first study was conducted in Chiang Mai in 2007 which revealed the detection rate of EV at 2.5% (Chaimongkol et al., 2012). However, this study did not identify the genotypes of the detected EV. In 2010–2014, another study conducted in Chiang Mai reported the prevalence of EV at 5.8%, of which CV-A24 and EV-C96 were the most prevalent genotypes detected (Kumthip et al., 2017). Another study was performed in Bangkok and Khon Kaen provinces located in the Central and Northeast of Thailand, respectively from 2010 to 2016 and the data revealed the detection rate of EV at 6.2%, of which PV-2 was the most common genotype (Chansaenroj et al., 2017). In the present study, the overall prevalence of EV infection from 2015 to 2018 was at 8.9%. The detection rates of EV in 2015, 2016, 2017, and 2018 were 7.2%, 9.0%, 11.2%, and 8.6%, respectively. The high prevalence of EV infection detected in 2017 may be due to an increase of some EV genotypes detection this year such as E9 which accounted for 19.4% of diarrheic cases. The detection rate of EV reported in this study (8.9%) is slightly higher than those of the previous studies (2.5–6.2%) conducted in Thailand, however, it is following the study from Vietnam in 2002 to 2003 which reported the prevalence of EV at 9.8% (Phan et al., 2005). The seasonality of EV infection has been demonstrated previously with the predominance in non-winter months during April–October (Rao et al., 2013) and June–August (Patil, Chitambar & Gopalkrishna, 2015). The present study clearly showed that EV infection occurred throughout the year with high detection rate during July–November which is corresponding to rainy season and the beginning of winter in Thailand. This finding is in line with the study conducted in India which reported that EV was most frequently detected in non-winter months (Patil, Chitambar & Gopalkrishna, 2015; Rao et al., 2013).

It has been reported that several EV genotypes, including E7, E11, E13, E30, and E33 are the common causes of acute flaccid paralysis and aseptic meningitis in children (Avellón et al., 2003; Rao, Yergolkar & Shankarappa, 2012; Zhao et al., 2005). The CV-A6, CV-A10, CV-A16, and EV-A71 were responsible for the outbreaks of hand-foot-and-mouth disease reported recently (Mirand et al., 2016; Rao et al., 2017; Wang et al., 2016; Weng et al., 2017; Xu et al., 2014). Also, some genotypes of EV have been reported to associate with acute gastroenteritis (Bina Rai et al., 2007; Rao et al., 2013; Patel, Daniel & Mathan, 1985; Patil, Chitambar & Gopalkrishna, 2015; Silva et al., 2008). The E11 and E30 have been found as the most prevalent genotypes associated with diarrhea in children in Southern India (Rao et al., 2013). Furthermore, the E6 and E14 were predominant in children with diarrhea in Western India from 2004 to 2009 (Patil, Chitambar & Gopalkrishna, 2015). The E11 was also responsible for an outbreak of acute gastroenteritis in confinement homes in Southern India (Patel, Daniel & Mathan, 1985) and Malaysia (Bina Rai et al., 2007). The study in Ghana reported that CV-A24 was the most predominant genotype identified in children with acute gastroenteritis (Silva et al., 2008). Although many studies reported the detection of EV in patients with gastroenteritis, the virus was also detected in asymptomatic individuals at the prevalence of 22.8% (Cristanziano et al., 2015).

Phylogenetic analysis based on the nucleotide sequence of the partial VP1 region revealed that EV strains detected in this study are genetically diverse. Four EV species (EV-A, EV-B, EV-C, and EV-D) and rhinovirus A were detected. Of 147 EV isolates detected in this study, 37 different EV genotypes were identified, of which CV-A24 and CV-B5 (7.5% each) were the most prevalent genotype, followed by EV-C96 (6.8%), etc. The results obtained from this study are in agreement with our previous study conducted in Thailand during 2010 to 2014 that the EV-C96 and CV-A24 are the most prevalent genotypes detected in patients with acute gastroenteritis (Kumthip et al., 2017), suggesting that the EV-C96 and CV-A24 are well spread and sustainable circulating in these patients. On the other hand, other EV genotypes, including E6, E14, and E30 were rarely detected while none of the E11 was detected in the present study. Moreover, this study could detect 12 strains of poliovirus and all of them were Sabin vaccine strains (97.6–100% nucleotide sequence identities). Other previous studies have also been detected the vaccine strains of poliovirus from the stool samples of diarrheic children. The presence of polio viral genomes in these fecal specimens is likely to be originated from oral polio vaccination (Chansaenroj et al., 2017; Kumthip et al., 2017; Rao et al., 2013; Shen et al., 2019). A minor adverse effects of oral polio vaccines (OPVs) such as headache, abdominal pain, fever, asthenia, and diarrhea have been documented. Approxiamately 10% of children who received OPV presented diarrhea at 1–9 days post-administration (Nzolo et al., 2013; Sugawara et al., 2009). In this study, the polio vaccine strains were detected in 12 out of 1,736 diarrheic cases (0.7%). It is possible that diarrhea in these patients may be resulted from the OPV vaccination. However, the possibility that diarrhea in these patients might cause either by other enteric viruses or bacteria was not ruled out. Furthermore, 28 EV strains detected in the present study are highly similar to EV strains detected previously in Thailand, suggesting that these EV strains are endemic in this area. In addition, several EV strains detected in this study were also closely related to EV strains reported previously from many other countries around the world, suggesting an effective spread of EV strains to several geographical regions worldwide.

The RV-A81, RV-A82, and EV-D68 have specific tissue tropism and usually cause the disease in the respiratory tract. Detection of these EV strains in the fecal specimens of diarrheic children in this study may reflect, at least in part, the adaptation of the virus in the gastrointestinal tract and sheds in the feces of infected patients. Detection of EV-D68 and RV in fecal specimens of diarrheic children have also been reported previously in Thailand (Chansaenroj et al., 2017). However, the potential role of EV-D68 and RV-A in the diarrheal disease is needed to be further elucidated.

In the present study, there are some limitations of the study. First, small numbers of stool samples were collected in some months. An equal number of samples tested each month is an ideal solution to determine the seasonal distribution of enterovirus infection study. Second, this study did not look for other enteric pathogens that cause acute gastroenteritis as the purpose of this study was only focused on the prevalence and diversity of EV in diarrheal patients. Third, this study did not include samples from the healthy control group. The significant association of enterovirus infection with gastrointestinal disease has been reported in a number of previous studies (Patil, Chitambar & Gopalkrishna, 2015; Rao et al., 2013; Rao, Maiya & Babu, 2014; Rao et al., 2014). The non-polio enterovirus (NPEV) infections were associated with acute and persistent diarrhea (18–21% of diarrheal cases) (Rao et al., 2013; Rao, Maiya & Babu, 2014) as well as increased frequency of bowel movements (approximately 29%) (Rao et al., 2014). Another study also revealed the evidence of some enterovirus strains induced severe diarhea in mouse model (Rao et al., 2015). In the case control study, the NPEV was more prevalent in patients with diarrhea (13.7–17%) than in healthy children (4.5–4.9%) (Patil, Chitambar & Gopalkrishna, 2015; Rao et al., 2013). The present study represented further confirmation of the role of enterovirus in acute diarrhea. Further studies of enterovirus infection in diarrheal patients and healthy control from different countries are still required to establish the impact of enterovirus with diarrhea.

## Conclusions

This study provides the epidemiological information, seasonal and genotypic distributions of human EVs circulating in children hospitalized with acute gastroenteritis in Chiang Mai, Thailand. The data may be useful for the implementation of the prevention and control measures for EV infection as well as the development of a vaccine against EV infection in the future.

##  Supplemental Information

10.7717/peerj.9645/supp-1 File S1 Enterovirus detection rate from January 2015 to December 2018

10.7717/peerj.9645/supp-2 File S2 Information of enterovirus strains detected in this study

10.7717/peerj.9645/supp-3 File S3 Nucleotide sequences of partial VP1 gene of enteroviruses detected in this study

## Additional Information and Declarations

### Data Availability

The following information was supplied regarding data availability: The raw data of enterovirus detection rate from January 2015 to December 2018 and the additional information and nucleotide sequences of enteroviruses are available in the Supplemental Files and the nucleotide sequences of the partial VP1 gene of enterovirus strains are available in GenBank: MN699137–MN699283.
