# Supplementary material for: Enterovirus infections in pediatric patients hospitalized with acute gastroenteritis in Chiang Mai, Thailand, 2015–2018
Source: PeerJ. 2020 Aug 17;8:e9645. doi: 10.7717/peerj.9645 (PMC7439955; doi:10.7717/peerj.9645)
Supplement: File S3 [file peerj-08-9645-s003.docx]

>CMH-S80-17

GCTAGCATCAACCATTTCTTTTCCCGTGCAGCTTTAGTGGGCAAGGTGGAATTAAACGAC

ACCGGCACCAGTGCCACAGGGTTCACCAACTGGGATATAGACATAATGGGATACGCGCAA

CTGCGCAGAAAATTGGAAATGTTCACTTACATGCGCTTCAACGCAAAGTTCACCTTTGTG

GCAACCACCAGGGCAGGGCAAGTGCCATCTAGAGTCCTCCAA

>CMH-ST54-17

GCTAGCATCAACCATTTCTTTTCCCGTGCAGCTTTAGTGGGCAAAGTGGAATTAAACGAC

ACCGGCACCAGTGCCACAGGGTTCACCAACTGGGATATAAACATAATGGGATACGCGCAA

CTGCGCAAAAAATTGGAAATGTTCACTTACATGCGCTTCAACGCAAAGTTCACCTTTGTG

GCAACCACCAGGGCAGGGCAAGTGCCATCTAAAGTCCTCCAA

>CMH-ST77-17

GCCAGCATAAACCACTTCTTTTCTCGTGCTGCTTTAGTTGGAAAGGTGGAACTAAATGAC

ACAGGCACGAGTGCTACAGGGTTCACAAATTGGGACATAGATATAATGGGGTATGCACAA

CTGCGTAGGAAACTGGAGATGTTCACGTACATGCGCTTCAACGCTGAGTTCACCTTCGTA

GCTACCACTAGGGCAGGGCAAGTGCCATCCAGGGTACTCCAA

>CMH-S165-16

ACTCATATTGAGCACTTCTTCTCTAGGTCAGGATTGGTGGGTATCATGGAGATAGATGAT

ACAGGCACTAGTGGCAAAGGATTCTCAAATTGGGATATCGACATCATGGCGTTTGTGCAA

TTGCGCCGTAAGCTTGAGGCATTCACATATATGCGATTTGACGCAGAGTTCACTTTTGTC

ACCAATCTGGAGAATGGGCTCACGAACAATAGTGTGATCCAG

>CMH-S185-16

ACCCATATTGAGCACTTCTTTTCTAGATCAGGATTGGTGGGTGTCATGGAGGTAGATGAT

ACAGGCACTAATGGAAAAGGATTCGCGAATTGGGACATTGACATCATGGCGTTTGTGCAG

TTGCGCCGCAAGCTTGAAGCATTCACATACATGCGGTTTGACGCAAAGTTCACCTTCGTC

ACTAACCTGGAAAATGGACTTACAAACAATAGTGTGATCCAA

>CMH-S199-16

ACCCATATCGAGCACTTCTTCTCTAGATCAGGATTGGTGGGTGTCATGGAGGTAGATGAT

ACAGGCACTAGTGGTAAAGGATTCTCGAACTGGGATATTGACATCATGGCGTTTGTGCAG

TTGCGCCGCAAGCTTGAAGCATTCACATACATGCGGTTTGACGCGGAGTTCACCTTCGTC

ACTAACCTGGAAAATGGGCTCACAAACAATAGTGTGATCCAA

>CMH-S243-16

ACTCATATTGAGCACTTCTTCTCTAGGTCAGGATTGGTGGGTGTCATGGAGATAGATGAT

ACAGGCACTAGTGGCAAAGGATTCTCAAATTGGGATATCGACATCATGGCGTTTGTGCAA

TTGCGCCGCAAACTTGAGGCATTCACATACATGCGGTTTGACGCAGAGTTCACTTTTGTC

ACCAATCTGGAGAATGGGCTCACGAACAATAGTGTGATCCAG

>CMH-ST157-16

ACTCATATTGAGCACTTCTTCTCTAGGTCAGGATTGGTGGGTGTCATGGAGATAGATGAT

ACAGGCACTAGTGGCAAAGGATTCTCAAATTGGGATATCGACATCATGGCGTTTGTGCAA

TTGCGCCGTAAACTTGAGGCATTCACATACATGCGGTTTGACGCAGAGTTCACTTTTGTC

ACCAACCTGGAGAATGGGCTCACGAATAATAGTGTGATCCAG

>CMH-S30-17

ACTCATATTGAGCACTTCTTCTCTAGGTCAGGGTTGGTGGGTGTCATGGAGATAGATGAT

ACAGGCACTAGTGGCAAAGGATTCTCAAATTGGGATATCGACATCATGGCGTTTGTGCAA

TTGCGCCGTAAACTTGAGGCATTCACATACATGCGGTTTGACGCAGAGTTCACTTTTGTC

ACCAATCTGGAGAATGGGCTCACGAACAATAGTGTGATCCAG

>CMH-S64-17

ACTCATATTGAGCACTTCTTCTCTAGGTCAGGATTGGTGGGTGTCATGGAGATAGATGAT

ACAGGCACTAGTGGCAAAGGATTCTCAAACTGGGATATCGACATCATGGCGTTTGTGCAA

TTGCGCCGTAAACTTGAGGCATTCACATACATGCGGTTTGACGCAGAGTTCACTTTTGTC

ACCAACCTGGAGAATGGGCTCACGAATAATAGTGTGATCCAG

>CMH-S115-18

ACTCATATTGAGCACTTCTTCTCTAGGTCAGGATTGGTGGGAGTCATGGAGATAGATGAT

TCAGGCACTAGTGGCAAAGGATTCTCAAATTGGGATATCGACATCATGGCGTTTGTGCAA

TTGCGCCGTAAACTTGAGGCATTCACATACATGCGGTTCGACGCAGAGTTCACTTTTGTC

ACCAATCTGGAGAATGGGCTCACGAACAATAGTGTGATCCAG

>CMH-S172-15

ACAAGTGTCGAGCACTTTTTCTCTCGTTCGGGCCTGGCAGGAATCTTGATAATTGAAGAC

TCTGGAACCTCTACAAAGGGGTATGCCACTTGGGAAATTGATGTGATGGGGTTTGTCCAA

TTGAGACGCAAGCTGGAAATGTTCACATACATGCGATTTGACGCAGAGTTCACCTTTATC

ACCGCGGAAAGGAACGGTAACACCAGTCCGATACCCGTCCAA

>CMH-N14-15

ACAAGTGTTGAGCACTTTTTCTCTCGTTCGGGCCTGGCAGGAATCTTGATAATTGAAGAT

TCTGGAACCTCTACAAAGGGGTATGCCACTTGGGAAATTGATGTGATGGGGTTTGTCCAA

TTGAGACGCAAGCTGGAAATGTTCACATACATGCGATTTGACGCAGAGTTCACCTTTATC

ACCGCGGAAAGGAACGGTAACACCAGTCCGATACCCGTCCAA

>CMH-ST174-18

ACAAGTGTTGAGCACTTTTTCTCTCGTTCGGGTCTGGCGGGAATCTTGATAATTGAGGAC

TCTGGAACTTCTACAAAGGGTTATGCCACTTGGGAAATTGATGTGATGGGATTTGTCCAA

TTGAGACGCAAGTTGGAAATGTTCACATACATGCGCTTTGACGCAGAGTTCACCTTTATC

ACTGCGGAAAGGAATGGTAACACCAGTCCGATACCCGTCCAA

>CMH-R139-18

ACAAGTGTTGAGCACTTTTTCTCTCGTTCGGGTCTGGCAGGAATCTTGATAATTGAGGAT

TCTGGAACTTCTACAAAAGGTTATGCCACTTGGGAAATTGATGTGATGGGATTTGTCCAA

TTGAGACGCAAGTTGGAAATGTTCTCATACATGCGATTTGACGCAGAGTTTACCTTTATC

ACAGCGGAAAGGAACGGTAACACCAGTCCGATACCCGTCCAA

>CMH-ST19-15

GCGAGTGTGGAACACTTTTACTCTCGTGCAGGGCTGGTAGGAGTTGTGGAGGTGAAGGAC

TCGGGCACTAGCCTGGATGGGTACACAGTTTGGCCCATAGATGTGATGGGCTTCGTGCAA

CAGCGGCGCAAGCTGGAACTGTCAACATACATGCGCTTTGATGCCGAGTTCACTTTTGTG

TCCAACCTCAATGACAGCACGACGCCCGGGATGCTGCTGCAG

>CMH-S73-16

GCGAGTGTGGAACACTTTTACTCTCGTGCAGGGCTGGTAGGAGTTGTGGAGGTGAAGGAC

TCGGGCACGAGCCTGGATGGATACACAGTTTGGCCTATAGATGTGATGGGCTTCGTGCAA

CAGCGGCGCAAGCTAGAGCTGTCAACATACATGCGCTTTGATGCCGAGTTCACTTTTGTG

TCCAACCTCAATGACAGCACGACGCCCGGGATGCTGCTACAG

>CMH-S193-16

GCGAGTGTGGAACACTTTTACTCTCGTGCAGGGCTGGTAGGAGTTGTGGAGGTGAAGGAC

TCGGGCACTAGCCCGGATGGGTACACAGTTTGGCCCATAGATGTGATGGGCTTCGTGCAA

CAGCGGCGCAAGCTAGAACTGTCAACGTACATGCGCTTTGATGCCGAGTTCACTTTTGTG

TCCAACCTCAATGACAGCACGACGCCCGGGATGCTGCTGCAG

>CMH-ST20-16

GCGAGTGTGGAACACTTTTATTCTCGTGCAGGGCTGGTAGGAGTTGTGGAGGTGAAGGAC

TCGGGCACTAGCCTGGATGGGTACACAGTTTGGCCCATAGATGTGATGGGCTTCGTGCAA

CAGCGGCGCAAGCTAGAACTGTCAACATACATGCGCTTTGATGCCGAGTTCACTTTTGTG

TCCAACCTCAATGACAGCACGACGCCCGGGATGCTGCTGCAG

>CMH-S114-15

ACCAGTTTGGACCACTTTTTTTCAAGGGCAGGTCTAGTAGGGGTGATCAATGTGCAAGAT

GGTGGTACACAGAAGGGATTCGAGGTGTGGGACGTAGACGTGATGGGATTTGTGCAGCTC

AGAAGGAAGTTGGAGATGTTTACGTACATGAGATTTAACGCTGAATTTACATTTGTGTCC

ACACTCGCAGACGGCACAACTCCCAGGGTGATGTTACAG

>CMH-N2-15

ACCAGTTTGGACCACTTCTTTTCAAGGGCAGGTCTAGTAGGGGTGATCAATGTGCAAGAT

GGTGGTACACAGAAGGGATTCGAGGTGTGGGACGTAGACGTGATGGGATTTGTGCAGCTC

AGAAGGAAGTTGGAGATGTTTACGTACATGAGATTTAACGCTGAATTTACATTTGTGTCC

ACACTCGCAGACGGCACAACTCCCAGGGTGATGTTACAG

>CMH-S164-18

ACTAGTCTAGATCACTTCTTCTCGCGTGCTGGGCTAGTAGGGGTAATCAACGTGCAAGAT

GGAGGTACTAATAAGGGCTACGAGGTTTGGGACATAGATGTGATGGGGTTCGTACAACTT

AGGAGGAAGTTGGAGATGTTTACATACATGAGATTTAATGCGGAATTCACATTTGTGTCA

ACTTTAGCTGACGGCACAACACCCAGAGTGATGCTGCAG

>CMH-ST184-18

ACTAGTCTAGATCACTTCTTCTCGCGTGCTGGGCTAGTAGGGGTAATCAACGTGCAAGAT

GGAGGTACTAATAAGGGCTACGAGGTTTGGGACATAGATGTGATGGGGTTCGTACAACTT

AGGAGGAAGTTGGAGATGTTTACATACATGAGATTTAATGCGGAATTCACATTTGTGTCA

ACTTTAGCTGACGGCACAACACCCAGAGTGATGCTGCAG

>CMH-S176-16

ACCACTATCAACCACTTCTTCTCCCGCTCTGGATTAGTGGGAGTAGTTAACCTCACAGAT

GGGGGGACAGACACCACTGGGTATGCCACTTGGGACATAGATATTATGGGCTTTGTCCAA

CTCCGCAGGAAATGCGAGATGTTCACATACATGAGGTTTAACGCAGAATTCACATTTGTC

ACAACGACTGAGAATGGAGAAGCTCGTCCGTACATGCTGCAA

>CMH-S177-16

ACCACTATCAACCACTTCTTCTCCCGCTCTGGATTAGTGGGAGTAGTTAACCTCACAGAT

GGGGGGACAGACACCACTGGGTATGCCACTTGGGACATAGATATTATGGGCTTTGTCCAA

CTCCGCAGGAAATGCGAGATGTTCACATACATGAGGTTTAACGCAGAATTCACATTTGTC

ACAACGACTGAGAATGGAGAAGCTCGTCCGTACATGCTGCAA

>CMH-S245-16

ACCACTATCAACCACTTCTTCTCCCGCTCTGGATTAGTGGGAGTAGTTAACCTCACAGAT

GGGGGGACAGACACCACTGGGTATGCCACTTGGGACATAGATATTATGGGCTTTGTCCAA

CTCCGCAGGAAATGCGAGATGTTCACATACATGAGGTTTAACGCAGAATTCACATTTGTC

ACAACGACTGAGAATGGAGAAGCTCGTCCGTACATGCTGCAA

>CMH-ST14-17

ACCACCATCAACCACTTCTTCTCCCGCTCTGGATTAGTGGGAGTAGTTAACCTCACAGAT

GGGGGGACAGACACCACTGGGTATGCCACTTGGGACATAGATATTATGGGCTTTGTCCAA

CTCCGCAGGAAATGCGAGATGTTCACATACATGAGGTTTAACGCAGAATTCACATTTGTC

ACAACGACTGAGAATGGGGAAGCTCGTCCGTACATGCTGCAA

>CMH-S181-18

ACTACTATCAATCACTTCTTTTCTCGCTCTGGTCTGGTGGGGGTAGTCAACCTCACTGAT

GGGGGGACTGATACTACAGGATATGCTACCTGGGATATAGACATTATGGGCTTTGTGCAG

CTCCGCAGGAAGTGTGAGATGTTTACGTATATGAGATTTAATGCTGAATTTACTTTTGTC

ACTACAACTGAGAATGGTGAAGCTCGCCCATACATGTTACAG

>CMH-ST242-18

ACAGCCATCGGGAATTTCTTTAGCCGTGCTGGTTTGGTTAGCATCATCACAATGCCCACC

ACGGGTACACAAAACACAAACGGTTACGTTAACTGGGATATTGACTTGATGGGATATGCT

CAGCTACGGCGCAAATGCGAGTTGTTTACGTATATGCCCTTTGATGCTGAATTCACATTT

GTCGTACCCAAACCTAATGGCGAATTGGTCCCCCAGTTACTGCAG

>CMH-N91-18

ACAGCCATCGGGAACTTCTTTAGCCGTGCTGGTTTGGTTAGCATCATCACAATGCCCACC

ACGGGTACACAGAATACCGACGGTTACGTTAACTGGGACATTGACTTGATGGGATATGCT

CAACTACGGCGCAAATGCGAGCTGTTTACGTACATGCGCTTTGATGCTGAATTCACATTT

GTCGTAGCCAAACCCAATGGCGAACTGGTCCCCCAGTTGCTGCAG

>CMH-N109-18

ACAGCCATCGGGAATTTCTTTAGCCGTGCTGGTTTGGTTAGCATCATCACAATGCCCACC

ACGGGTACACAGAACACAGACGGTTACGTTAACTGGGATATTGACTTGATGGGATATGCT

CAGCTACGGCGCAAATGCGAGTTGTTTACGTATATGCGCTTTGATGCTGAATTCACATTT

GTCGTACCCAAACCCAATGGCGAATTGGTCCCCCAGTTACTGCAG

>CMH-S115-17

ACCACCCTGGACAGTTTCTTCAGCAAAGCAGGTTTGGTAGGAGAGATAGATCTTCCACTA

AAGGGTACCACCAACCCAAATGGTTATGCTAACTGGGATATAAACATAACTGGTTATGCG

CAAATGCCCAGGAAAGTGAAGCTGTTCACCTATATGCGCTTTGATGCGAAGTTCACTTTC

GTTGCGTGCACTCCTACTGGTGAGGTTGTTCCGCAATTACTCCAGTATATGTTCGTCCCC

CCAGGTGGTCCAGTA

>CMH-S98-15

CGGTCGGAGTCAACAATTGAGAACTTCCTTGCCAGGTCAGCATGTGTCTTTTTCCTAGAG

TACAAAACTGGCACCAAAGAGGACTCTAATAGCTTCAACAATTGGGTGATCACCACGAGA

CGAGTGGCCCAACTACGCAGGAAATTGGAAATGTTCACCTACTTGAGGTTTGACATGGAG

ATCACTGTGGTTATCACAAGTTCGCAGGACCAATCAACGTCACAGAATCAGAATGCCCCT

GTGCTGACACAT

>CMH-ST186-18

AGGACCGAGTCGTCTATTGAAAACTTCCTGTGCAGGGCCGCATGTGTGTACATAACAACG

TACAAATCAGCCGGGGAGACGCCAACAGAAAGATATGCAAGTTGGCGCATTAACACTAGA

CAGATGGTACAACTCAGGAGGAAGTTCGAGCTCTTTACCTACCTGCGTTTTGACATGGAG

ATCACTTTTGTCATCACCAGCACACAAGACCCTGGGACCCGCTTAGCGCAGGACATGCCT

GTTTTGACGCAC

>CMH-ST198-18

AGGACCGAGTCGTCTATTGAAAACTTCCTGTGCAGGGCCGCATGTGTGTACATAACAACG

TACAAATCAGCCGGGGGGACGCCAACAGAAAGATATGCAAGTTGGCGCATTAACACTAGA

CAGATGGTACAACTCAGGAGGAAGTTCGAGCTCTTTACCTACCTGCGTTTTGACATGGAG

ATCACTTTTGTCATCACCAGCACACAAGACCCTGGGACCCAGTTAGCGCAGGACATGCCT

GTTTTGACGCAC

>CMH-S111-16

AGATCAGAATCCACTATAGAGAACTTTTTAAGTAGGTCTGCGTGTGTGCACATAGCCAAT

TACAACGCGAAGGGAGACAAAACAGATGTCAACAGGTTTGATAGATGGGAGATCAACATC

CGTGAGATGGTGCAGTTGCGAAGAAAGTGCGAGATGTTTACTTACCTGCGCTTTGACATT

GAGGTAACATTTGTGATAACCAGCAAGCAAGATCAGGGAACCAACCTTAACCAGGATATG

CCAGTGCTCACTCAT

>CMH-S152-16

AGATCAGAATCCACTATAGAGAACTTTTTAAGTAGGTCTGCGTGTGTGCACATAGCCAAT

TACAACGCGAAGGGAGACAAAACAGATGTCAACAGGTTTGATAGATGGGAGATCAACATC

CGTGAGATGGTGCAGTTGCGAAGAAAGTGCGAGATGTTTACTTACCTGCGCTTTGACATT

GAGGTAACATTTGTGATAACCAGCAAGCAAGATCAGGGAACCAACCTTAACCAGGATATG

CCAGTGCTCACTCAT

>CMH-S196-16

AGATCAGAATCCACTATTGAAAATTTCTTAAGCAGAGCAGCATGTGTGCACATAGCTAAC

TACAATGCAAAGGGTGATAAGACAGATGTCAACAGATTTGACAGGTGGGAAATCAATATT

CGTGAGATGGTGCAACTACGCAGGAAGTGTGAGATGTTCACGTACTTGCGCTTCGACATC

GAGGTGACGTTCGTGATAACCAGCAAACAAGATCAGGGAACCAACTTAAACCAAGATATG

CCAGTCCTCACACAC

>CMH-ST123-16

AGATCAGAATCCACTATTGAAAATTTCTTAAGCAGAGCAGCATGTGTGCACATAGCTAAC

TACAATGCAAAGGGTGATAAGACAGATGTCAACAGATTTGACAGGTGGGAAATCAATATT

CGTGAGATGGTGCAACTACGCAGGAAGTGTGAGATGTTCACTTACTTGCGCTTCGACATC

GAGGTGACGTTCGTGATAACCAGCAAACAAGATCAGGGAACCAACTTAAACCAAGATATG

CCAGTCCTCACTCAC

>CMH-S250-15

AGATCGGAATCGTCCGTGGAGAACTTCCTCAGTAGATCTGCCTGTGTCTACATTGTAGAA

TACAAAACAAGAGACGATACCCCAGACAAAATGTATGATAGTTGGGTTATAAACACTAGG

CAAGTGGCTCAGTTGCGCAGGAAGCTGGAGTTCTTCACGTATGTGAGATTTGACGTGGAA

GTTACATTTGTTATAACCAGTGTGCAGGATGATTCAACTAGGCAGAATACTGACACGCCA

GCACTCACACAC

>CMH-S190-16

AGATCAGAATCGTCCGTGGAGAACTTCCTCAGTAGATCTGCCTGTGTTTACATTGTAGAA

TACAAAACAAGAGACGATACCCCAGACAAAATGTATGATAGTTGGGTTATAAACACTAGG

CAAGTGGCTCAGTTGCGCAGGAAGCTGGAGTTCTTCACGTATGTGAGATTTGATGTGGAA

GTTACATTTGTTATAACCAGTGTGCAGGATGATTCAACTAGGCAGAATACTGACACGCCA

GCACTCACACAC

>CMH-S198-16

AGATCAGAATCGTCCGTGGAGAATTTCCTCAGTAGATCTGCCTGTGTTTACATTGTAGAA

TACAAAACAAGAGACGATACCCCAGACAAAATGTATGATAGTTGGGTTATAAACACTAGG

CAAGTGGCTCAGTTGCGCAGGAAGCTGGAGTTCTTCACGTATGTGAGATTTGATGTGGAA

GTTACATTTGTTATAACCAGTGTGCAGGATGATTCAACTAGGCAGAATACTGACACGCCA

GCACTCACACAC

>CMH-ST78-16

AGATCAGAATCGTCCGTGGAGAACTTCCTCAGTAGATCTGCCTGTGTTTACATTGTGGAA

TACAAAACAAGAGACGATACCCCAGACAAAATGTATGATAGTTGGGTTATAAACACTAGG

CAAGTGGCTCAGTTGCGCAGGAAGCTGGAGTTCTTCACGTATGTGAGGTTTGATGTAGAA

GTTACATTTGTTATAACCAGTGTGCAGGATGATTCAACTAGGCAGAATACCGACACGCCA

GCACTCACACAC

>CMH-ST106-16

AGATCAAAATCGTCCGTGGAGAACTTCCTCAGTAGATCTGCCTGTGTTTACATTGTAGAA

TACAAAACAAGAGACGATACCCCAAACAAAATGTATGATAGTTGGGTTATAAACACTAGG

CAAGTGGCTCAGTTGCGCAGGAAGCTGGAGTTCTTCACGTATGTGAGGTTTGATGTAAAA

GTTACATTTGTTATAACCAGTGTGCAGGATGATTCAACTAGGCAGAATACAGACACGCCA

GCACTCACACAC

>CMH-S104-17

CGGTCCGAGTCCACCATAGAGAACTTCCTGTGCAGGGCGGCTTGTGTGCGCATGGCAAAA

TATCAGGCAAGAGGCGACCCCGAGAGTACTGACCGCTTTGATGCGTGGGAGATAAGTATA

CGAGACATGGTCCAATTGCGCCGCAAGTGTGAGATGTTCACATACTTGCGCTTTGATGTG

GAAGTCACATTTGTAATAACTAGTTACCAGCATCAGGGGACCATTAACCAAGACATGCCC

CCAATGACCCAC

>CMH-S124-17

CGGTCTGAATCCACCGTAGAAAACTTCCTGTGTAGGGCGGCTTGTGTGCGTATGGCCAAA

TATGAAGCAAGGGGCGATCCTGAGAGCACCGATCGCTTTGATGCATGGGAGATAAGCATA

CGTGACATGGTTCAACTGCGCCGCAAGTGCGAGATGTTTACATACTTGCGCTTTGACGTG

GAAGTTACGTTTGTGATAACTAGTTACCAACACCAAGGGACTATCAACCAGGACATGCCC

CCAATGACTCAC

>CMH-ST57-17

CGGTCCGAGTCCACCATAGAGAACTTCCTGTGCAGGGCAGCTTGTGTGCGCATGGCAAAA

TATCAGGCAAGAGGCGACCCCGAGAGTACTGACCGCTTTGATGCGTGGGAGATAAGTATA

CGAGACATGGTCCAATTGCGCCGCAAGTGTGAGATGTTCACATACTTGCGCTTTGATGTG

GAAGTCACATTTGTAATAACTAGTTACCAGCATCAGGGGACCATTAACCAAGACATGCCC

CCAATGACCCAC

>CMH-N10-17

CGGTCTGAGTCCACCGTAAAAAACTTCCTGTGTAGGGCGGCCTGTGTGCGTATGGCCAAA

TATGAAGCAAGGGGCGATCCTGAGAGCACCGATCGCTTTGATGCATGGGAGATAAGCATA

CGTGACATGGTTCAAATGCGCCGCAAGTGCGAGATGTTTACATACTTGCGCTTTGATGTG

GAAGTTACATTTGTGATAACTAGTTACCAGCACCAGGGGACTATCAACCAAGACATGCCC

CCGATGACTCAT

>CMH-N16-17

CGGTCCGAGTCCACCATTGAGAACTTCCTGTGCAGGGCAGCTTGTGTGCGCATGGCAAAA

TATCAGGCAAGAGGCAACCCCGAGAGTACTGACCGCTTTGATGCATGGGAGATAAGTATA

CGAGACATGGTCCAATTGCGCCGCAAGTGTGAGATGTTCACATACTTGCGCTTTGATGTG

GAAGTCACATTTGTAATAACTAGTTACCAGCATCAAGGGACCATTAACCAAGACATGCCC

CCAATGACCCAC

>CMH-N27-17

CGGTCCGAGTCCACCATTGAGAACTTCCTGTGCAGGGCAGCTTGTGTGCGCATGGCAAAA

TATCAGGCAAGAGGCGACCCCGAGAGTACTGACCGCTTTGATGCATGGGAGATAAGTATA

CGAGACATGGTCCAATTGCGCCGCAAGTGTGAGATGTTCACATACTTGCGCTTTGATGTG

GAAGTCACATTTGTAATAACTAGTTACCAGCATCAAGGGACCATTAACCAAGACATGCCC

CCAATGACCCAC

>CMH-ST54-18

CGGACCGAGTCCACCATTGAGAACTTCCTGTGCAGGGCAGCTTGTGTGCGCATGGCAAAA

TATCAGGCAAGAGGCAACCCCGAGAGTACTGACCGCTTTGATGCATGGGAGATAAGTATA

CGAGACATGGTCCAATTGCGCCGCAAGTGTGAGATGTTCACATACTTGCGCTTTGATGTG

GAAGTCACATTTGTAATAACTAGTTACCAGCATCAAGGGACCATTAACCAAGACATGCCC

CCAATGACCCAC

>CMH-N12-16

AGGTCTGAATCATCAGTTGAGAACTTTCTGTGTCGATCTGCGTGTGTATACTACACTACT

TACGACACACATGGTGACGGAGCAGACCAGAAGTATGCCAGTTGGACCATTACAACCCGT

AAAGTGGCCCAGTTGCGCAGAAAACTTGAGATGTTCACCTACCTAAGATTCGACCTGGAG

GTGACGTTCGTCATAACGAGTGCGCAAGTGACCTCCACAAACCAAAGTCAGGACGCACCC

GTACTCACCCAT

>CMH-S122-17

CGTTCAGAATCATCCATAGAAAATTTCTTGTGCCGCTCGGCTTGTGTGTATTACACAACG

TATGACACACACGGGGACGCTGCAAATGCTAAGTACGCTAGTTGGACTATCACAACGCGT

AAGGTTGCTCAATTGAGGAGAAAGCTGGAAATGTTCACATACTTAAGATTTGATTTGGAA

GTGACGTTTGTTATTACAAGCGCCCAAGTCACCTCCACAAATCAAAGTCAAGATGCACCC

GTTCTTACCCAC

>CMH-S197-15

AGGTCAGAGTCAACCATAGAAAACTTCTTGTGTAGGTCTGCATGCGTGCACATAGCCACC

TACAAAGCAAAGGGTAGTGCTGGAGACACCAATCGTTACGACAGTTGGGATATCAACATT

AAGGAGTTGGTGCAGCTTCGGAGAAAGTGCGAGATGTTCACCTACCTAAGGTTTGACATG

GAGGTTACATTTGTGATAACCAGCATACAGGAGCAGGGCACGGCCTTGACACAGGACATG

CCAGTTTTGACCCAC

>CMH-ST111-16

AGGTCAGAGTCAACTATAGAGAACTTCTTGTGTAGATCTGCGTGTGTGCACATAGCCACT

TACAAAGCAAAGGGTGGTGCTGGAGATACCGAACGTTATGACAGCTGGGATATTAACATT

AAGGAGTTAGTACAGCTCCGGAGAAAGTGTGAGATGTTTACCTACCTGAGATTCGATATG

GAGGTCACTTTTGTAATAACCAGCATCCAGGAACAGGGCACGGCTTTAACACAGGACATG

CCAGTGTTGACCCAC

>CMH-ST118-16

AGGTCAGAGTCAACTATAGAGAACTTCTTGTGTAGATCTGCGTGTGTGCACATAGCCACT

TACAAAGCAAAGGGTGGTGCTGGAGATACCGAACGTTATGACAGCTGGGATATTAACATT

AAGGAGTTAGTACAGCTCCGGAGAAAGTGTGAGATGTTTACCTACCTGAGATTCGATATG

GAGGTCACTTTTGTAATAACCAGCATCCAGGAACAGGGCACGGCTTTAACACAGGACATG

CCAGTGTTGACCCAC

>CMH-S134-18

AGGACTGAATCAACACTAGAGAATTTTCTTGGGAGATCAGCATGCGTGCATATCGACACA

TACAAGGCAAAGGGTGAGAAAGGTTCTTCTGAAAGGTATGCCAGCTGGGAAATAACAACT

AGGGAGATGGTCCAGCTGCGTCGTAAGTGTGAGATGTTCACGTATATGCGATATGACGTG

GAGGTGACCTTCGTAATCACTAGCTACCAAGAACAGAGCACACAATTAACCCAAGACATG

CCAGTTTTAACGCAT

>CMH-ST211-18

AGATCAAAGTCCACTATAGAAAACTTTTTGTGTAGGTCAGCATGCGTGCACATAGCCAAG

TACGAGGCCAGTGCGAACGCAAATAATGAAGATAGATTTGTGAGGTGGGAAATAAACAAC

AAAGAATTAGTCCAGCTTAAAAGGAAGTGTGAAATGTTCACGTACCTTCGCTATGACGTG

GAGGTTACTTTTGTGATCACTAGTCAACAAGATCAAGGAACAGACTTATCCCAAGATATG

CCAGTGCTTACCCAC

>CMH-N95-18

AGATCAAAGTCCACTATAGAAAACTTTTTGTGTAGGTCAGCATGCGTGCACATAGCCAAG

TACGAGGCCAGTGCGAACGCAAATAATGAAAATAGATTTGTGAGGTGGGAAATAAACAAC

AAAGAATTAGTCCAGCTTAAAAGGAAGTGTGAAATGTTCACGTACCTTCGCTATGACGTG

GAGGTTACTTTTGTGATCACAAGTCAACAAGATCAAGGAACAGACTTATCCCAAGATATG

CCAGTGCTTACCCAC

>CMH-S19-15

CGATCTGAGTCCACCGTGGAAAACTTCATGGGGCGTGCTGCCTGCGTGTTCATGGACCAA

TATAAGTTAAACGGTGAGGAAACATCCACCGACAACTTCGCAGTATGGACCATAAACATT

AGGGAGATGGCACAGTTAAGGAGGAAGTGTGAAATGTTCACGTACATGCGTTTTGATATT

GAGATGACAATGGTGATTACTAGCTGCCAAGATCAAGGGACGCAGTTGGAGCAAGACATG

CCCGTCTTGACGCAC

>CMH-S208-16

CGATCCGAGTCTACCGTAGAGAACTTCATGGGGCGTGCCGCCTGCGTGTTCATGGACCAA

TACAAGTTAAATGGAGAGGAAACATCCACCGACAACTTCGCAGTGTGGACAATAAACGTT

AGGGAAATGGCACAGTTAAGGAGGAAGTGCGAAATGTTCACATACATGCGTTTTGATATT

GAGATGACAATGGTAATCACTAGTTGCCAAGATCAAGGGACGCAGTTGGAGCAAGATATG

CCTGTCTTGACTCAC

>CMH-ST198-16

CGATCCGAGTCCACCGTGGAAAACTTCATGGGGCGTGCCGCCTGCGTGTTCATGGACCAA

TACAAGTTAAATGGAGAGGAAACATCCACCGACAATTTCGCAGTGTGGACAATAAACGTT

AGGGAAATGGCACAGTTAAGGAGGAAGTGCGAAATGTTCACATACATGCGTTTTGATATT

GAGATGACAATGGTGATCACTAGCTGCCAAGATCAAGGGACGCAGTTGGAGCAAGATATG

CCTGTCTTGACGCAC

>CMH-S89-17

AGATCTGAGTCCAGCATTGAGAACTTCTTGAGTAGGTCGGCCTGCGTGTATATGGGTGAG

TATAGTACAAAGGCTTCAGAAGAGACAAAGAAGTACATGTCTTGGACCATCAGCCCTAGA

AGAATGGTTCAAATGCGCAGGAAGTTTGAACTTTTCACATACCTGCGCTTTGACGTGGAA

GTCACCTTTGTTATCACTAGCAGACAGGATGAAGCCGGGACACAATATGGTCAAGACGCG

CCACCTCTGACCCAT

>CMH-N108-18

AGATCTGAGTCCAGCATTGAAAATTTCCTGAGCAGGTCAGCCTGTGTGTACATGGGTGAA

TATAGCACAAAAGCCTCGGAAGAGACAAAGAAGTACATGTCTTGGACCATCAGCCCTAGA

AGGATGGTTCAAATGCGCAGAAAGTTTGAACTCTTCACATACTTGCGCTTTGATGTAGAA

ATCACTTTTGTCATCACCAGTAGACAAGACGAGGCCGGGACGCAATATGGACAAGATGCG

CCACCCCTGACCCAT

>CMH-S126-18

CGGTCCGAATCAACAATTGAAAATTTCATGTGCAGGGCGGCGTGCGTGTACATCGCTCGA

TATGGCACTGACAAGCAAGGGGAGCAGATCTCCAGACATACAAAATGGAAAATCACTACA

CGGCAGGTGGCTCAATTAAGGAGAAAAATGGAAATGTTCACATACATGAGGTTTGACCTG

GAGATGACTTTTGTGATCACAAGCTCACAGCGCACGTCTACAACATATGATTCGGACACG

CCAGTTTTGACACAT

>CMH-ST244-18

CGGTCCGAATCAACAATTGAAAATTTCATGTGCAGGGCGGCGTGCGTGTACATCGCTCGA

TATGGCACTGACAAGCAAGGGGAGCAAATTTCCAGATATACAAAATGGAAAATCACTACG

CGGCAGGTGGCTCAATTAAGGAGAAAAATGGAAATGTTCACATACATGAGGTTTGACCTG

GAGATGACTTTTGTGATCACAAGCTCACAGCGCACGTCTACAACATATGATTCGGACACG

CCAGCTTTGACACAT

>CMH-ST249-18

CGGTCCGAATCAACAATTGAAAATTTCATGTGCAGGGCGGCGTGCGTGTACATCGCTCGA

TATGGCACTGACAAGCAAGGGGAGCAGATTTCCAGATATACAAAATGGAAAATCACTACG

CGGCAGGTGGCTCAATTAAGGAGAAAAATGGAAATGTTCACATACATGAGGTTTGACCTG

GAGATGACTTTTGTGATCACAAGCTCACAGCGCACGTCTACAACATATGATTCGGACACG

CCAGCTTTGACACAT

>CMH-S14-15

AGGTCAGAATCTTCAATAGAAAATTTTCTGAGTAGATCAGCATGTGTCTACATTGATGTA

TATAGCACAAAAGAGAATGGCGACATCAAACGCTTTACCAATTGGAAAATCAACACACGC

CAGGTTGTTCAACTAAGGCGCAAACTGGAGATGTTCACTTACATCAGATTTGATGTGGAA

ATAACATTTGTGATTACGAGCACCCAAGGGACGTCAACCCAAACAAGCACAGACACCCCA

GTGCTCACACAT

>CMH-S30-15

AGGTCAGAATCTTCAATAGAAAATTTTCTGAGTAGATCAGCATGTGTCTACATTGATGTA

TATAGCACAAAAGAGAATGGCGACATCAAACGCTTTACCAATTGGAAAATCAACACACGC

CAGGTTGTTCAACTAAGGCGCAAACTGGAGATGTTCACTTACATCAGATTTGATGTGGAA

ATAACATTTGTGATTACGAGCACCCAAGGGACGTCAACCCAAACAAGCACAGACACCCCA

GTGCTCACACAT

>CMH-S40-18

AGGTCAGAATCATCGATTGAGAACTTTCTGAGCAGATCGGCATGTGTCTACATTGACGTC

TATAGTACAAAAGAGAATGGTGACATTAAACGCTTCACCAACTGGAAAATCAATACGCGT

CAGGTTGTCCAACTAAGGCGCAAGCTGGAGATGTTCACGTATATCAGATTTGATGTGGAG

GTTACATTTGTGATAACAAGCACTCAAGGGACGTCAACCCAAACAAGCACAGACACTCCG

GTACTCACACAC

>CMH-S117-18

AGGTCAGAATCATCGATTGAGAACTTTCTGAGCAGATCGGCATGTGTCTACATTGACGTC

TATAGCACAAAAGAGAATGGTGACATTAAACGCTTCACCAACTGGAAAATCAATACGCGT

CAGGTTGTCCAACTAAGGCGCAAGCTGGAGATGTTCACGTATATCAGATTTGATGTGGAG

GTTACATTTGTGATAACAAGCACTCAAGGGACGTCAACCCAAACAAGCACAGACACTCCG

GTACTCACACAC

>CMH-ST116-18

AGGTCAGAGTCATCGATTGAGAACTTTTTGAGTAGATCGGCATGTGTCTATATTGACGTC

TATAGCACAAAAGAGAACGGTGACATCAAACGCTTCACCAACTGGAAAATTAACACGCGT

CAGGTTGTCCAACTAAGGCGCAAACTGGAGATGTTCACGTATATCAGATTTGATGTGGAG

GTTACGTTTGTGATAACGAGCACTCAAGGGACGTCAACCCAAACAAACACAGACACCCCA

GTACTCACACAT

>CMH-ST153-18

AGGTCAGAATCATCGATTGAGAACTTTCTGAGCCGATCGGCATGTGTCTACATTGACGTC

TATAGTACAAAAGAGAATGGTGACATTAAACGCTTCACCAACTGGAAAATCAATACGCGT

CAGGTTGTCCAACTAAGGCGCAAGCTGGAGATGTTCACGTATATCAGATTTGATGTGGAG

GTTACATTTGTGATAACAAGCACTCAAGGGACGTCAACCCAAACAAGCACAGACACTCCG

GTACTCACACAC

>CMH-R74-18

AGGTCAGAATCATCGATTGAGAACTTTCTGAGCAGGTCGGCATGTGTCTACATTGACGTC

TATAGTACAAAAGAGAATGGTGACATTAAACGCTTCACCAACTGGAAAATCAATACGCGT

CAGGTTGTCCAACTAAGGCGCAAGCTGGAGATGTTCACGTATATCAGATTTGATGTGGAG

GTTACATTTGTGATAACAAGCACTCAAGGGACGTCAACCCAAACAAGCACAGACACTCCG

GTTCTCACACAC

>CMH-ST55-17

AGATCAGAGTCATCAATTGAAAACTTCATGGGTAGGGCGGCGTGTGTGTATATCGCCCAG

TACGCCACTGAGAAAGTCAATGATGAGTTGGACAGGTACACTAACTGGGAGATAACAACC

AGGCAAGTGGCACAGCTAAGGCGCAAGCTGGAAATGTTCACATACATGAGATTCGACCTC

GAGATCACGTTAGTCATCACCAGCTCTCAACGCACCTCAACCACATACGCATCAGACTCC

CCACCACTGACACAC

>CMH-N37-17

AGATCAGAGTCATCAATTGAAAACTTCATGGGTAGGGCGGCGTGTGTGTATATCGCCCAG

TACGCCACTGAGAAAGTCAACGATGAGTTGGACAGGTACACTAACTGGGAGATAACAACC

AGGCAAGTGGCACAGCTAAGGCGCAAGCTGGAAATGTTCACATACATGAGATTCGACCTC

GAGATCACGTTAGTCATCACCAGCTCTCAACGCACCTCAACCACATACGCATCAGACTCC

CCACCACTGACACAC

>CMH-S191-15

CGATCCGAATCCACCGTGGAAAATTTTCTTGGTAGATCCGCTTGCGTATACATGGAGGAA

TACAAGACCACGGATAGTGATACCAATAAGAAATTCGTAGCGTGGCCAATTAACACCAAA

CAAATGGTGCAAATGCGCAGAAAGCTAGAGATGTTTACCTACCTCAGGTTTGACATGGAG

GTGACCTTTGTGATCACAAGCCGGCAGGACCCTGGGACCACACTAGCACAAGACATGCCA

GTACTCACGCAC

>CMH-N54-16

CGATCCGAATCCACCGTGGAAAATTTTCTTGGTAGATCCGCTTGTGTATACATGGAAGAA

TACAAGACCACGGATAGTGATACCAATAAGAAGTTCGTAGCGTGGCCAATTAACACCAAA

CAAATGGTGCAAATGCGCAGAAAGCTAGAGATGTTTACCTACCTCAGGTTTGACATGGAG

GTGACTTTTGTGATCACAAGTCGGCAGGACCCTGGGACCACACTAGCACAAGACATGCCA

GTACTCACGCAC

>CMH-ST197-18

CGTTCTGAATCCACCGTGGAAAACTTTCTAGGCAGGTCAGCTTGTGTCTATATGGAGGAG

TACAAGACCACAGACAATGATGTTAACAAGAAATTTGTGGCGTGGCCGATCAATACTAAA

CAAATGGTGCAAATGCGTAGGAAGCTAGAGATGTTCACCTACCTTAGGTTTGACATGGAA

GTAACTTTTGTGATCACAAGCCGGCAAGATCCTGGGACCACATTAGCACAAGACATGCCA

GTGCTAACGCAC

>CMH-S170-18

AGATCTGAATCAACCATAGAGAACTTCTTGTGTCGATCCGCTTGTGTTTACTATGCCACC

TACACAAACAACACAGAAAAAGGGTATGCAGAGTGGGTCGTGAACACTAGGCAGGTAGCC

CAATTGAGGAGAAAGCTGGAGCTGTTCACCTACTTAAGATTTGATTTAGAGTTGACATTT

GTGATAACGAGCGCCCAACAACCCAGCACTGCCACTAGTGTGGACGCCCCTGTACAAACG

CAC

>CMH-ST169-18

AGATCTGAATCAACCATAGAGAACTTCTTGTGTCGATCCGCTTGTGTTTACTATGCCACC

TACACAAACAACACAGAAAAAGGGTATGCGGAGTGGGTCGTGAACACTAGGCAGGTAGCC

CAATTGAGGAGAAAGCTGGAGCTGTTCACCTACTTAAGATTTGATTTAGAGCTGACATTT

GTGATAACGAGCGCCCAACAACCCAGCACTGCCACTAGTGTGGACGCCCCTGTACAAACG

CAC

>CMH-N92-18

AGATCTGAATCAACCATAGAGAACTTCTTGTGTCGATCCGCTTGTGTTTACTATGCCACC

TACACAAACAACACAGAAAAAGGGTATGCAGAGTGGGTCGTGAACACTAGGCAGGTAGCC

CAATTGAGGAGAAAGCTGGAGCTGTTCACCTACTTAAGATTTGATTTAGAGTTGACATTT

GTGATAACGAGCGCCCAACAACCCAGCACTGCCACTAGTGTGGACGCCCCTGTACAAACG

CAC

>CMH-S205-16

AGATCTGAGTCGACAATTGAGAACTTCGTGTGCAGGTCCGCGTGTGTTTATTTCACAGAG

TACGAGAACTCGGGATCAAATCGGTATGCTGAATGGGTGATAACAACTCGCCAAGCAGTA

CAGTTGAGAAGAAAGTTGGAGTTCTTCACATACATGAGGTTTGATTTAGAGCTCACCTTC

GTTATTACGAGTACTCAGCAACCCTCTACAACCCAGAACCAAGACGCCCAAATTCTCACC

CAT

>CMH-N73-18

AGATCTGAGTCGACAATTGAAAACTTCGTGTGCAGGTCCGCGTGCGTTTATTTTACAGAG

TACGAGAACTCGGGGTCAAATCGGTATGCTGAATGGGTAATAACAACTCGCCAAGCAGTA

CAGTTGAGAAGAAAGTTGGAGTTCTTCACATACATGAGGTTTGATTTAGAGCTCACCTTC

GTTATCACGAGTACTCAGCAACCCTCTACAACCCAGAACCAAGACGCCCAAATTCTCACC

CAT

>CMH-R131-18

AGATCTGAGTCGACAATTGAGAACTTCGTGTGCAGGTCCGCGTGCGTTTATTTTACAGAG

TACGAGAACTCGGGGTCAAATCGGTATGCTGAATGGGTAATAACAACTCGCCAAGCAGTA

CAGTTAAGAAGAAAGTTGGAGTTTTTCACATACATGAGGTTTGATTTAGAGCTCACCTTC

GTTATCACGAGTACTCAGCAACCCTCTACAACCCAGAACCAAGACGCCCAAATTCTCACC

CAT

>CMH-ST30-16

AGATCAAAGTCATCTATAGAAAACTTTCTATGCAGGTCCGCTTGTGTAATCTACATAAAG

TATTCTAGCGCTGAGTCAAACAAATTGAAGCGTTATGCAGAGTGGGTCATCAACACAAGA

CAGGTAGCTCAGCTGAGGCGCAAGATGGAAATGTTCACTTACATCCGGTGTGACATGGAA

CTCACCTTTGTAATCACCAGCCACCAGGAAATGTCCACCGCCACTAACTCGGATGTGCCA

GTGCAAACGCAC

>CMH-S35-15

AGATCGGAGTCGACGGTGGAGAACTTCCTGTGTAGATCAGCATGCGTCTTTTATACCACA

TATAGAAATCATGGTACCGACGGTGACAACTTTGGTTATTGGGTGATCAGCACGCGTCAG

GTGGCTCAACTACGGCGCAAGCTTGAGATGTTTACATATGCGAGATTTGATCTCGAGCTA

ACCTTTGTGATCACGAGCACTCAAGAACAGTCTACCATACAAGGCCAGGATTCACCAGTA

CTCACACAT

>CMH-S173-15

AGATCGGAGTCGACGGTAGAGAACTTTCTGTGTAGATCAGCATGCGTCTTTTACACCACA

TATAGAAATCATGGTACTGATGGTGACAACTTTGGTTATTGGGTGATCAGCACGCGCCAG

GTGGCTCAACTACGGCGCAAGCTTGAGATGTTTACATATGCGAGATTTGATCTCGAGCTA

ACCTTTGTGATCACAAGCACTCAAGAGCAGTCCACCATACAAGGCCAGGATTCACCAGTG

CTCACACAT

>CMH-N5-15

AGATCGGAGTCGACGGTAGAGAACTTTCTGTGTAGATCAGCATGCGTCTTTTACACCACA

TATAGAAATCATGGTACTGATGGTGACAACTTTGGTTATTGGGTGATCAGCACGCGCCAG

GTGGCTCAACTACGGCGCAAGCTTGAGATGTTTACATATGCGAGATTTGATCTCGAGCTA

ACCTTTGTGATCACAAGCACTCAAGAGCAGTCCACCATACAAGGCCAGGATTCACCAGTG

CTCACACAT

>CMH-N9-15

AGATCGGAGTCGACGGTAGAGAACTTTCTGTGTAGATCAGCATGCGTCTTTTACACCACA

TATAGAAATCATGGTACTGATGGTGACAACTTTGGTTATTGGGTGATCAGCACGCGCCAG

GTGGCTCAACTACGGCGCAAGCTTGAGATGTTTACATATGCGAGATTTGATCTCGAGCTA

ACCTTTGTGATCACAAGCACTCAAGAGCAGTCCACCATACAAGGCCAGGATTCACCAGTG

CTCACACAT

>CMH-N15-15

AGATCGGAGTCAACAGTGGAGAACTTTCTGTGTAGATCAGCATGTGTCTTTTACACCACG

TATAAAAACCATGGTACTGACGGTGATAACTTTGGTTATTGGGTGATCAGCACGCGCCAG

GTGGCCCAACTGCGGCGCAAACTTGAGATGTTTACATATGCGAGATTCGATCTTGAGCTG

ACCTTTGTGATCACGAGTACACAAGAACAGTCCACTATACAAGGTCAGGATTCACCAGTA

CTTACGCAT

>CMH-N33-15

AGATCGGAGTCAACAGTGGAGAACTTTCTGTGTAGATCAGCATGTGTCTTTTACACCACG

TATAAAAACCATGGTACTGACGGTGATAACTTTGGTTATTGGGTGATCAGCACGCGCCAG

GTGGCCCAACTGCGGCGCAAACTTGAGATGTTTACATATGCGAGATTCGATCTTGAGCTG

ACCTTTGTGATCACGAGTACACAAGAACAGTCCACTATACAAGGTCAGGATTCACCAGTA

CTTACGCAT

>CMH-S53-18

AGATCGGAGTCAACGGTGGAGAACTTCCTGTGTAGATCAGCATGCGTCTTTTACACCACG

TATAGAAATCATGGTACCGACGGTGATAACTTTGGTTATTGGGTGATCAGCACGCGTCAA

GTGGCTCAACTAAGGCGCAAGCTTGAGATGTTTACATATGCGAGATTTGACCTCGAGCTA

ACCTTTGTGATCACGAGCACTCAAGAACAGTCTACCATACAAGGCCAGGATTCACCAGTA

CTCACACAT

>CMH-ST136-18

CGATCTGAATCCACAGTAAAGAACTTTCTGTGTAGATCCGCGTGTGTGTATTACACAACT

TACAAGAACCATGGTACTGATGGAGACAATTTCGCCTACTGGGTGATCAACACAAGACAG

GTCGCGCAGTTGCGCCGCAAATTAGAAATGTTCACGTATGCCAGATTTGACTTAGAACTC

ACATTTGTGATAACAAGCACACAAGAACAATCCACTATCCAAGGTCAAGACTCGCCCGTG

TTAACTCAT

>CMH-ST162-18

CGATCTGAATCCACAGTAGAGAACTTTCTGTGTAGATCCGCGTGTGTGTATTACACAACT

TACAAGAACCATGGTACTGATGGAGACAATTTCGCCTACTGGGTGATCAACACAAGACAG

GTCGCGCAGTTGCGCCGCAAATTAGAAATGTTCACGTATGCCAAATTTGACTTAGAACTC

ACATTTGTGATAACAAGCACACAAGAACAATCCACTATCCAAGGTCAAGACTCGCCCGTG

TTAACTCAT

>CMH-N105-18

CGATCTGAATCCACAGTAAAGAACTTTCTGTGTAGATCCGCGTGTGTGTATTACACAACT

TACAAAAACCATGGCACTGATGGAGACAATTTCGCCTACTGGGTGATCAACACAAAACAG

GTCGCGCAGTTGCGCCGCAAATTAAAAATGTTCACGTATGCCAAATTTGACTTAAAACTC

ACATTTGTGATAACAAGCACACAAAAACAATCCACTATCCAAGGTCAAAACTCGCCCGTG

TTAACTCAT

>CMH-R69-18

AGATCGGAGTCAACGGTGGAGAACTTCCTGTGTAGATCAGCATGCGTCTTTTACACCACG

TATAGAAATCATGGTACCGACGGTGACAACTTTGGTTATTGGGTGATCAGCACGCGTCAA

GTGGCTCAACTAAGGCGCAAGCTTGAGATGTTTACATATGCGAGATTTGATCTCGAGCTA

ACCTTTGTGATCACGAGCACTCAAGAACAGTCTACCATACAAGGTCAGGATTCACCAGTA

CTCACACAT

>CMH-S59-15

AGGAGCGAATCCACCATTGAAAGCTTCTTCGGGCGATCCGCCTGTGTCACAATAATGTCA

GTGGAAAATTTCAATGCTACTGAAATGTCAGATAAGAAAAGACTGTTTAGTGTGTGGGAA

ATCAGTTACACAGACACTGTCCAGCTTAGGCGGAAGTTAGAAATGTTCACATACTCGCGC

TTCGACACGGAGTTCACTTTCGTGTTGACCGAGAGGTACTACACCCAGAACTCAGGCCAC

GCTCGCAACCAGGTCTATCAAATC

>CMH-ST36-16

AGGAGTGAATCCACCATTGAAAGCTTCTTCGGGCGATCCGCCTGTGTCACAATAATGTCA

GTGGAAAATTTCAATGCTACTGACACAGCAGACAAGAAAAAACTGTTTAGTGTGTGGGAA

ATTAGCTACACAGACACTGTCCAGCTCAGGCGGAAGTTAGAAATGTTCACATACTCGCGT

TTCGACACTGAATTCACTTTCGTGTTGACCGAGAGGTATTACACCCAAAACTCAGGCCAC

GCTCGCAACCAGGTCTATCAGATC

>CMH-ST77-16

AGGAGTGAATCAACCATTGAGAGTTTCTTTGGGCGGTCAGCTTGCATTACAATAATGTCC

GTGGAAAACTTCAATGCTACCGAGTCAGCAGACAAAAAGAAACTGTTTAGCGTGTGGGAG

ATCAACTACACAGACACAGTCCAACTCAGACGAAAATTGGAGATGTTCACATACTCACGC

TTTGACACAGAGTTCACTTTCGTGTTGACTGAGAGATATTATACCCAAAACTCAGGACAT

GCTCGTAATCAGGTCTATCAAATC

>CMH-ST94-16

AGGAGTGAATCCACCATTGAAAGCTTCTTTGGGCGATCCGCCTGTATCACAATAATGTCA

GTGGAAAATTTCAACGCTACTGAAGTAGCGGACAAGAGAAAGCTGTTTAGTGTGTGGGAA

ATCGGTTACACGGATACTGTCCAGCTTAGACGAAAGTTAGAAATGTTCACATACTCGCGT

TTTGACACGGAGTTCACTTTCGTGTTGACCGAGAGGTACTACACCCAGAACTCAGGCCAC

GCTCGCAACCAGGTCTATCAAATT

>CMH-ST165-16

AGAAGTGAATCAACCATTGAGAGTTTCTTTGGGCGGTCACCTTGCATTACAATAATGTCT

GTGGAAAATTTCAATGCTACCGATACGACAGATAAAAAGAAACTGTTTAGTGTGTGGGAG

ATCAACTATACAGACACAGTCCAACTCAAACGCAAATTGGAGATGTTCACATACTCACGC

TTTGACACAGAGTTCACTTTTGTGCTGACTGATAGATATTACACCCAAAATTCAGGACAT

GCTCGTAATCAGGTCTACCAAATC

>CMH-S150-18

AGAAGTGAATCGACCATTGAAAGCTTCTTTGGGCGGGCGGCTTGCATCACTATAATGTCA

GTGGAGAATTTCAATGCTACTGAAAGTTCAGACAAGAAGAAGTTGTTCAGTGTATGGGAG

ATTAGCTACACAGATACAGTCCAACTCAGACGAAAATTGGAGATGTTCACATATTCACGC

TTTGACACAGAGTTCACTTTCGTTTTGACAGAAAGATATTACACCCAGAACTCAGGACAT

GCTCGCAATCAGGTTTACCAAATT

>CMH-ST2-18

AGGAGTGAATCAACCATTGAAAGCTTCTTTGGGCGATCTGCCTGCATCACGATAATGTCA

GTAGAAAATTTCAATGCCACTGAAATGTCAGACAAAAAGAAACTGTTCAGTGTGTGGGAA

ATCAATTACACAGATACTGTCCAACTCAGGCGGAAGTTAGAGATGTTCACATACTCGCGT

TTTGACACGGAATTTACTTTTGTGTTAACCGAGAGATACTACTCCCAGAATTCAGGTCAC

GCCCGCAATCAGGTTTACCAGATC

>CMH-ST38-18

AGGAGTGAATCCACCATTGAGAGCTTCTTCGGGCGATCCGCCTGTGTCACGATAATGTCA

GTGGAAAATTTTAATGCTACTGAATCAACAGACAAGAAAAAACTGTTTAGTGTGTGGGAA

ATTAGCTACACAGACACTGTCCAGCTCAGGCGGAAGTTAGAAATGTTCACATACTCGCGC

TTCGACACTGAACTCACTTTCGTGTTGACCGAGAGGTATTACACCCAAAATTCAGGCCAC

GCCCGCAACCAGGTCTATCAGATC

>CMH-ST73-18

AGAAGTGAGTCAACCATTGAAAGCTTCTTTGGGCGGTCAGCTTGCATTACAATAATGTCA

GTGGAAAACTTCAACGCTATTGAAACGGTAGACAAGAAAAAATTATTCAGTGTATGGGAG

ATCAATTACACAGATACTGTCCAACTTAGACGAAAATTAGAGATGTTCACATACTCGCGC

TTTGACACAGAGTTCACTTTCGTATTGACTGAAAGATATTACACCCAAAACTCAGGGCAT

GCTCGTAATCAGGTTTACCAAATC

>CMH-ST216-18

AGGAGTGAATCAACCATTGAAAGCTTCTTTGGGCGATCTGCCTGCATCACGATAATGTCA

GTAGAAAATTTTAATGCCACTGATACGTCAGACAAAAAGAGACTGTTCAGTGTGTGGGAA

ATCAATTACACAGATACTGTCCAACTCAGGCGGAAGTTAGAGATGTTCACATACTCGCGT

TTTGACACGGAATTTACTTTTGTGTTAACCGAAAGATACTACTCCCAGAATTCAGGTCAC

GCCCGCAATCAGGTTTACCAGATC

>CMH-S188-15

AGGAGTGAGAGCACAGTAGAGTCCTTCTTTGCTCGAGGCGCATGTGTGGCAATAATGAGT

GTAGATAACTACAATGAAACACTCACTAGTAATCAAAAATCCACTTTATTCGCCACTTGG

AACATCACCTACACTGATACTGTGCAACTAAGAAGAAAATTAGAAATGTTTACATATTCC

AGATTTGACATCAAATTCACTTTTGTGGTAACCGAGCGCTACTTCTCAAGTAATGCAGGA

CATGCATTAAATCAGGTGTATCAAATA

>CMH-N10-15

AGGAGTGAGAGCACAGTAGAGTCCTTCTTTGCTCGAGGGGCATGTGTGACAATAATGGGT

GTAGATAACTACAATGAAACACTCACTGGTAATCAAAAATCCACTTTATTCGCCACTTGG

AACATCACCTACACTGATACTGTGCAACTAAGAAGAAAATTAGAAATGTTTACATATTCC

AGATTTGACATCGAATTCACTTTTGTGGTAACCGAGCGCTACTACTCCAGTAATGCAGGA

CATGCATTAAACCAGGTATATCAAATC

>CMH-N29-15

AGGAGTGAAAGCACAGTAAAGTCCTTCTTTGCTCGAGGCGCATGTGTGACGATAATGGGT

GTAGATAACTACAATGAAACACTCACTGGTAATCAAAAATCCACTTTATTCGCCACTTGG

AACATCACCTACACTGATACTGTGCAGCTAAGAAGAAAATTGGAAATGTTTACATACTCC

AGATTTGACATCGAATTCACTTTTGTGGTAACCGAGCGCTATTACTCCGGTAATGCAGGA

CATGCATTAAATCAGGTGTATCAAATA

>CMH-N37-15

AGGAGTGAGAGCACAATAGAGTCCTTCTTTGCTCGAGGGGCTTGTGTGACAATAATGGGT

GTAGAAAATTACAATGAAACACTCACGGGAAACCAAAAATCCACTTTTTTTGCCACTTGG

AACATAACCTACACTGATACTGTCCACCTAAGAAGGAAATTAGAGATGTTTACTTATTCC

AGATTTGACATCGAATTCACTTTTGTGGTAACGGAGCGCTACTATTCAGCCAATGGCGGC

CATGCCCTAAACCAAGTTTATCAAATC

>CMH-ST148-16

AGGAGTGAGAGCACAGTGGAATCCTTCTTTGCTCGAGGGGCTTGTGTGACAATAATGGGT

GTGGATAATTACAATGAAAAACTCACGGATGGCCAAAAATCCACTTTATTTGCCACTTGG

AACATAACCTACACTGATACTGTGCAACTAAGAAGAAAATTAGAAATGTTTACTTATTCC

AGATTTGACATTGAATTCACTTTTGTGGTAACGGAGCGCTACCATTCAACTGGTGCAGGA

AATGCCCTAAACCAGGTATATCAAATA

>CMH-N33-17

AGGAGTGAGAGCACAGTAGAGTCCTTCTTTGCTCGAGGTGCATGTGTGACGATAATGAGT

GTAGAAAACTACAATGAAACTCTCACTGGTAGTCAGAAATCCACCTTATTCGCCACTTGG

AACATCACCTATACTGACACTGTGCAGCTAAGAAGAAAATTGGAAATGTTCACATACTCC

AGATTTGATATCGAATTCACTTTTGTAGTAACCGAGCGTTACCATTCCAGCAATTCAGGA

CACGCACTCAACCAGGTGTATCAAATC

>CMH-ST64-18

AGGAGTGAGAGCACGGTGGAGTCCTTCTTTGCACGAGGGGCGTGTGTCACCATTATGAGT

GTTGATAATTACAACGAGACACTCACTGGTAATCAAAGGTCAACACTATTTGCAACTTGG

AATATTACTTACACTGACACTGTACTGCTCAGAAGAAAACTAGAAATGTTTACATACTCC

AGATTTGATATAGAGTTCACTTTTGTTGTGACTGAAAGCTACTACTCAAGCAATGCTGGA

CACTCATTGAACCAAGTGTATCAAATA

>CMH-ST193-18

AGAAGTGAAAGCACAGTGGAATCTTTCTTTGCTCGAGGAGCATGCGTGACAATAATGGGA

TTGGACAATTATAACGAAACACTAACTAACGGTCAAAAGTCCACACTGTTTGCAACTTGG

AACATCACTTATACTGACACTGTACAATTGAGAAGAAAACTAGAAATGTTCACATACTCC

AGATTTGACTTTGAATTTACATTTGTGGTAACTGAGCGTTACTACTCAACCAACTCAGGA

CACGCACTAAACCAGGTCTATCAAATC

>CMH-ST67-17

AGATCAGAGTGCACTGTAGAATCCTTCTTTGGGAGATCTGCTTGTGTAGCTATAATAGGC

TTGAGTAATAAGAAGCCCACAGACACCAATGGGAAAGAACTGTTTGCAACATGGCCTATT

TCATACTTGGACACATACCAGCTAAGGAGAAAGTTAGAGATGTTTACTTATTCCAGGTTT

GACATTGAAATGACCTTTGTTATAACAGAAAGATTCTTCACCTCAACATCTGCGGCTGCT

AGAGACTATGTATATCAGATC

>CMH-ST69-17

AGATCAGAGTGCACTGTAGAATCCTTCTTTGGGAGATCTGCTTGTGTAGCTATAATAGGC

TTGAGTAATAAGAAGCCCACAGACACCAATGGGAAAGAACTGTTTGCAACATGGCCTATT

TCATACTTGGACACATACCAGCTAAGGAGAAAGTTAGAGATGTTTACTTATTCCAGGTTT

GACATTGAAATGACCTTTGTTATAACAGAAAGATTCTTCACCTCAACATCTGCGGCTGCT

AGAGACTATGTATATCAGATC

>CMH-N11-17

AGATCAGAGTGCACTGTAGAATCCTTCTTTGGGAGATCTGCTTGTGTAGCTATAATAGGC

TTGAGTAATAAGAAGCCCACAGACACCAATGGGAAAGAACTGTTTGCAACATGGCCTATT

TCATACTTGGACACATACCAGCTAAGGAGAAAGTTAGAGATGTTTACTTATTCCAGGTTT

GACATTGAAATGACCTTTGTTATAACAGAAAGATTCTTCACCTCAACATCTGCGGCTGCT

AGAGACTATGTATATCAGATC

>CMH-ST218-18

AGATCAGAGTGCACTGTAGAGTCCTTCTTTGGGAGATCTGCTTGTGTGGCTATAATAGGC

TTGAGTAATAAGAAGCCCACAGACACTAATGGGAAAGATCTGTTTGCAACATGGCCTATT

TCATACTTGGACACATACCAACTAAGGAGAAAGCTAGAGATGTTTACTTACTCCAGGTTT

GACATTGAAATGACCTTTGTTATAACAGAAAGATTCTTCACCTCAACATCTGCAGCAGCT

AGGGACTACGTATATCAGATC

>CMH-S99-17

AGGTCTGAGTCATGTCTTGAATCATTCTTTGGGAGAGCTGCGTGTGTCACAATCCTATCT

CTGACCAACTCTTCACAAAGTGGAGAGGAAAAGAAACATTTCAACATTTGGAACATCACG

TATACCGACACTGTGCAGTTACGTAGAAAATTAGAGTTCTTCACATATTCCAGATTTGAC

CTTGAAATGACTTTTGTGTTCACAGAGAACTACCCCAGTACAGCTAGTGGAGAAGCGCGC

AACCAGGTATACCAGATC

>CMH-N41-17

AGGTCTGAGTCATGTCTTGAATCATTCTTTGGGAGAGCTGCGTGTGTCACAATCCTATCT

CTGACCAACTCTTCAGAAAGTGGAGAGGAAAAGAAACATTTCAACATTTGGAACATCACG

TATACCGACACTGTGCAGTTACGTAGAAAATTAGAGTTCTTCACATATTCCAGATTTGAC

CTTGAAATGACTTTCGTGTTTACAGAGAACTACCCCAGTACAGCTAGTGGAGAAGTGCGC

AACCAGGTATACCAGATT

>CMH-N48-15

AGATCCGAATCAACGCTTGAATCTTTCTTTGGTAGATCAGCATGTGTCACCATTCTTGAA

GTCGAGAATTTTAATGCAACCACCGTGCAAGATAAAAAGAAGCAATTCACAACCTGGCCA

ATCACGTATACCGACACTGTGCAGTTACGCAGAAAATTAGAGTTCTTCACTTATTCTAGA

TTTGACTTAGAGATGACTTTTGTGGTGACTGAAAGGTATTATACTTCAAACACAGGACAC

GCCAGAAACCAAGTTTACCAGATA

>CMH-S121-16

AGGTCTGAATCGACACTTGAATCCTTCTTTGGTAGATCAGCTTGTGTCACCATCCTCGAG

GTTGAGAATTTCAACGCCACTACTGAAACAGACAAAAGGAAGCAATTTACGACTTGGCCC

ATTACATATGCCAATACTGTGCAATTGCGCAGGAAGCTAGAATTTTTCACTTACTCCAGA

TTTGATTTGGAGATGACCTTCGTGGTGACTGAGAGGTTTTACGCTAGCAATACAGGACAC

GCCAGGAATCAGGTTTATCAATTA

>CMH-ST26-16

AGGTCTGAATCAACACTGGAATCCTTCTTTGGCAGATCAGCCTGTGTCACTATCCTTGAG

GTTGAGAATTTCAACGCAACCGATGCAGCGGACAAAAGAAAACAATTTACAATTTGGCCG

ATCACATACACCAACACCGTGCAGTTGCGCAGGAAGTTAGAATTCTTCACTTATTCCAGA

TTTGATCTAGAAGTGACTTTTGTGATAACTGAAAGGTACTACACTAGCAACTCAGGACAC

GCCAGAAACCAGGTTTATCAAATA

>CMH-ST34-16

AGATCTGAGTCTACACTTGAATCCTTCTTCGGCAGATCAGCATGTGTCACAATTCTAGAA

GTTGAAAACTTCAATGCAACTACTACAGAAGACAAAAAGAAACAGTTTACCACCTGGTCT

ATCACATATACTGACACTGTCCAGTTACGTAGGAAATTGGAATTCTTCACTTACTCCAGA

TTTGACCTAGAGATGACTTTTGTGGTGACTGAGAGGTATTACACCAGCAACACCGGACAT

GCTAGGAACCAGGTTTACCAGATC

>CMH-ST122-16

AGATCAGAATCCACACTGGAATCCTTCTTTGGTAGGTCAGCGTGTGTCACGATCATTGAA

GTTGAAAATTTTAATGCAACTACCATTGAAGACAAAAAGAAACAGTTCGCCGTCTGGCCA

ATCACATACACCAATACTGTCCAACTACGAAGGAAGTTAGAATTTTTCACTTACTCCAGA

TTTGATCTAGAGATGACTTTTGTAGTGACTGAGAGATATTACACCTCCAACACCGGTCAT

GCCAGAAATCAGGTGTACCAGATT

>CMH-ST185-16

AGGTCTGAATCCACACTGGAGTCTTTCTTTGGTAGATCAGCGTGTGTTACGATCATTGAA

GTTGAAAATTTTAATGCAACTACCGCAGATGACAAAAAGAAACAGTTTACCACCTGGCCA

ATCACATACACTGACACTGTCCAACTGCGTAGGAAGTTGGAATTTTTCACTTACTCCAGA

TTTGACTTAGAGATGACTTTTGTAGTGACTGAGAGATATTACACCAGCAACTCCGGCCAT

GCCAGAAATCAAGTCTACCAGATC

>CMH-ST49-17

AGATCAGAATCCACCCTGGAATCTTTCTTTGGTAGGTCTGCTTGTGTTACAATATTGGAG

GTTGAAAATTTTAACGCCACTACCGCAGCAGACAAGAGGAGGCAATTCACCATATGGCCC

ATCACGTACACCAACACTGTGCAATTACGCAGGAAACTTGAGTTCTTCACATACTCCAGG

TTTGACTTAGAAATGACTTTTGTAGTAACTGAAAGGTATTACGCATCAAACACAGGACAT

GCTAGAAATCAGGTATACCAAATA

>CMH-ST76-17

AGGTCTGAATCCACACTTGAATCCTTCTTCGGCAGGTCAGCATGTGTCACAATTTTGGAA

GTGGAAAATTTCAATGCAACCACCGAAAATGACAAAAAGAAACAGTATACCATTTGGCCC

ATTACATACACCAACACTGTCCAACTGCGTAGAAAGCTAGAGTTTTTCACCTACTCCAGA

TTTGATCTAGAAATGACTTTTGTGGTGACTGAAAGATATTACACCAGTAACACTGGCCAT

GCTAGGAATCAGGTATACCAGATT

>CMH-N5-17

AGGTCTGAATCCACACTAGAGTCTTTCTTTGGTAGATCAGCGTGTGTCACGATCATTGAA

GTTGAAAATTTCAATGCAACTACCACGGATGATAAAAAGAAACAGTTTGCTACCTGGCCA

ATCACGTACACTGACACTGTCCAACTGCGTAGGAAGTTGGAATTTTTCACTTACTCCAGA

TTTGATCTAGAGATGACCTTTGTGGTGACTGAGAGATATTATACCAGCAACACCGGCCAT

GCCAGAAATCAGGTCTACCAGATC

>CMH-N21-17

AGGTCTGAATCCACATTGGAGTCTTTCTTTGGCAGATCAGCGTGTGTTACGATCATTGAA

GTTGAAAATTTTAATGCAACTACCGCAGATGACAAAAAGAAACAGTTTGCCACCTGGCCA

ATCACATACACTGACACTGTCCAACTACGTAGGAAGTTGGAATTTTTCACTTACTCCAGA

TTTGACTTAGAGATGACTTTTGTGGTGACTGAGAGATACTACACCAGCAACACTGGCCAT

GCCAGAAATCAAGTCTACCAGATC

>CMH-S63-18

AGGTCTGAATCCACGCTTGAATCCTTCTTTGGCAGGTCAGCCTGTGTCACAATTCTTGAA

GTTGAAAATTTCAATGCAACCACTGAAAGTGACAGGAAAAAACTATTCACCACATGGCCC

ATTACATACACCAACACTGTCCAGTTACGAAGGAAGCTAGAATTTTTCACTTACTCTAGA

TTTGACTTAGAAATGACTTTTGTAGTGACTGAGAGATATTATACCAGCAACTCCGGCCAT

GCCAGAAATCAGGTATATCAAATT

>CMH-S62-16

CGATCAGAGTCCACGGTTGAGTCATTCTTTGCAAGAGGGGCTTGCGTGGCTATCATTGAG

GTGGACAATGATGCACCGACAAAGCGCGCCAGCAGATTGTTTTCGGTTTGGAAAATAACT

TACAAAGATACTGTTCAACTGAGACGCAAACTGGAATTTTTCACATATTCGAGATTTGAC

ATGGAGTTCACTTTTGTGGTCACCTCAAACTACATTGATGCAAATAACGGACATGCATTG

AACCAAGTTTATCAGATA

>CMH-S124-16

CGATCAAAGTCCACGGTTGAGTCATTCTTTGCAAGAGGGGCTTGCGTGGCTATCATTGAG

GTGGACAATGATGCACCGACAAAGCGCGCCAGCAGATTGTTTTCGGTTTGGAAAATAACT

TACAAAGATACTGTTCAACTGAGACGCAAACTGGAATTTTTCACATATTCGAGATTTGAC

ATGGAGTTCACTTTTGTGGTCACCTCAAACTACACTGATGCAAATAACGGACATGCATTG

AACCAAGTTTATCAAATA

>CMH-ST35-16

CGATCAAAGTCCACGGTTGAGTCATTCTTTGCAAGAGGGGCTTGCGTGGCTATCATTGAG

GTGGACAATGATGCACCGACAAAGCGCGCCAGCAGATTGTTTTCGGTTTGGAAAATAACT

TACAAAGATACTGTTCAACTGAGACGCAAACTGGAATTTTTCACATATTCGAGATTTGAC

ATGGAGTTCACTTTTGTGGTCACCTCAAACTACGTTGATGCAAATAACGGACATGCATTG

AACCAAGTTTATCAAATA

>CMH-S157-16

AGGTCAGAGTCCACAATAGAATCATTCTTCGCACGCGGGGCGTGCGTCGCTATTATTGAG

GTGGACAATGAACAACCAACCACCCGGGCACAGAAACTATTTGCCATGTGGCGCATTACA

TACAAAGATACAGTGCAGTTGCGCCGTAAGTTGGAGTTTTTCACATACTCTCGTTTTGAC

ATGGAATTCACCTTCGTGGTAACCGCCAACTTCACCAACGCTAATAATGGGCATGCACTC

AACCAGGTGTACCAGATA

>CMH-ST189-16

AGGTCAGAGTCCACAATAGAATCATTCTTCGCACGCGGGGCGTGCGTCGCTATTATTGAG

GTGGACAATGAACAACCAACCACCCGGGCACAGAAACTATTTGCCATGTGGCGCATTACA

TACAAAGATACAGTGCAGTTGCGCCGTAAGTTGGAGTTTTTCACATACTCTCGTTTTGAC

ATGGAATTCACCTTCGTGGTAACCGCCAACTTCACCAACGCTAATAATGGGCATGCACTC

AACCAGGTGTACCAGATA

>CMH-N30-17

AGGTCAGAGTCCACAATAGAATCATTCTTCGCACGCGGGGCGTGCGTCGCTATTATTGAG

GTGGACAATGAACAACCAACCACCCGGGCACAGAAACTATTTGCCATGTGGCGCATTACA

TACAAAGATACAGTGCAGTTGCGCCGTAAGTTGGAGTTTTTCACATACTCTCGTTTTGAC

ATGGAATTCACCTTCGTGGTAACCGCCAACTTCACCAACGCTAATAATGGGCATGCACTC

AACCAGGTGTACCAGATA

>CMH-ST51-18

AGGTCAGAGTCCACAATAGAATCATTCTTCGCACGCGGGGCGTGCGTCGCTATTATTGAG

GTGGACAATGAACAACCAACCACCCGGGCACAGAAACTATTTGCCATGTGGCGCATTACA

TACAAAGATACAGTGCAGTTGCGCCGTAAGTTGGAGTTTTTCACATACTCTCGTTTTGAC

ATGGAATTCACCTTCGTGGTAACCGCCAACTTCACCAACGCTAATAATGGGCATGCACTC

AACCAGGTGTACCAGATA

>CMH-ST60-18

AGGTCAGAGTCCACAATAGAATCATTCTTCGCACGCGGGGCGTGCGTCGCTATTATTGAG

GTGGACAATGAACAACCAACCACCCGGGCACAGAAACTATTTGCCACGTGGCGCATTACA

TACAAAGATACAGTGCAGTTGCGCCGTAAGTTGGAGTTTTTCACATACTCTCGTTTTGAC

ATGGAATTCACCTTCGTGGTAACCGCCAACTTCACCGACGCTAATAATGGGCATGCACTC

AACCAGGTGTACCAGATA

>CMH-ST210-18

AGGTCAGAGTCCACAATAGAATCATTCTTCGCACGCGGGGCGTGCGTCGCTATTATTGAG

GTGGACAATGAACAACCAACCACCCGGGCACAGAAACTATTTGCCATGTGGCGCATTACA

TACAAAGATACAGTGCAGTTGCGCCGTAAGTTGGAGTTTTTCACATACTCTCGTTTTGAC

ATGGAATTCACCTTCGTGGTAACCGCCAACTTCACCAACGCCAATAATGGGCATGCACTC

AACCAGGTGTACCAGATA

>CMH-N68-18

AGGTCAGAGTCCACAATAGAATCATTCTTCGCACGCGGGGCGTGCGTCGCTATTATTGAG

GTGGACAATGAACAACCAACCACCCGGGCACAGAAACTATTTGCCATGTGGCGCATTACA

TACAAAGATACAGTGCAGTTGCGCCGTAAATTGGAGTTTTTCACATACTCTCGTTTTGAC

ATGGAATTCACCTTCGTGGTAACCGCCAACTTCACCAACGCTAATAATGGGCATGCACTC

AACCAGGTGTACCAGATA

>CMH-N90-18

AGGTCAGAGTCCACAATAGAATCATTCTTCGCACGCGGGGCGTGCGTCGCTATTATTGAG

GTGGACAATGAACAACCAACCACCCGGGCACAGAAACTATTTGCCATGTGGCGCATTACA

TACAAAGATACAGTGCAGTTGCGCCGTAAGTTGGAGTTTTTCACATACTCTCGTTTTGAC

ATGGAATTCACCTTCGTGGTAACCGCCAACTTCACCAACGCTAATAATGGGCATGCACTC

AACCAGGTGTACCAGATA

>CMH-N98-18

AGGTCAGAGTCCACAATAGAATCATTCTTCGCACGCGGGGCGTGCGTCGCTATTATTGAG

GTGGACAATGAACAACCAACCACCCGGGCACAGAAACTATTTGCCATGTGGCGCATTACA

TACAAAGATACAGTGCAGTTGCGCCGTAAGTTGGAGTTTTTCACATACTCTCGTTTTGAC

ATGGAATTCACCTTCGTGGTAACCGCCAACTTCACCAACGCTAATAATGGGCATGCACTC

AACCAGGTGTACCAGATA

>CMH-N34-17

ACTCGCACAGTTATAAATCAACACGGTGTATCCGAAACTCTAGTGGAGAATTTTCTCAGT

AGAGCAGCTTTGGTATCAAAGAGAAGTTTCGAATACAAAGACCATACTTCGTCTGCAGCA

CAAGCAGATAAGAACTTTTTCAAATGGACAATTAACACCAGGTCCTTCGTACAGCTAAGA

AGAAAACTAGAATTATTCACATACCTTAGATTCGATGCTGAGATCACTATACTCACAACT

GTAGCAGTGAATGGTAGCAGTAATAATGCATATGTGGGTCTTCCTGACTTGACACTGCAG

GCAATGTACGTGCC

>CMH-ST78-17

CAATCCTTACAAACACTGGATGAGATGAGTGTAGAGAGTTTCTTAGGTAGGGCAGGCTGT

ATTCATGAATCTATACTGGATATCAAAGAGGATTATAACACTCAAAGTTTTACTAAGTGG

AAAATCAATTTACAGGAGATGGCACAAATTAGAAGAAAATTTGAAATGTTTACATATACC

AGGTTTGATTCTGAGATCACATTAGTACCAAGTATTGCAAACAAAGAAGGTCACATTGGT

CACATAGTCATGCAATACATGTACGTG

>CMH-S74-17

CAAACATCACAAACCAGGGATGAAATGAGTCTTGAGAGTTTCCTAGGGAGATCTGGTTGC

ATACACATATCACACTTAGAAGTTAAATATACTGATTATAATGAAAGTAATAATTTTAGA

TCATGGCCAATAAGTATAAAAGAGATGGCACAAATAAGAAGAAAGTTTGAGCTTTTCACG

TATGTGAGATTTGATTCAGAAATCACTCTGGTGCCTTGCATAGCCTCTCAAAGTAGTGAT

ATTGGACATGTAGTGATGCAGTACATGTTCGTG
